# Korean medicine registry for cognitive disorder: A protocol for prospective observational multi-center study

**DOI:** 10.1371/journal.pone.0323170

**Published:** 2025-05-15

**Authors:** Hanbit Jin, Do-Eun Lee, Moon Joo Cheong, Hyungsun Jun, Taena Eom, Seojae Jeon, Dong-Hoon Kang, Hye-Jeong KooK, Daeun Lee, In Chul Jung, Jungtae Leem, Hyung Won Kang

**Affiliations:** 1 Department of Diagnostics, College of Korean Medicine, Wonkwang University, Iksan, Republic of Korea; 2 Department of Korean Neuropsychiatry Medicine, College of Korean Medicine, Wonkwang University, Iksan, Republic of Korea; 3 Department of Medical Counseling, College of Health Sciences, Wonkwang University, Iksan, Republic of Korea; 4 Department of Digital Healthcare, Graduate School of JABA, Wonkwang University, Iksan, Republic of Korea; 5 Department of Neuropsychiatry, College of Korean Medicine, Daejeon University, Daejeon, Republic of Korea; 6 Clinical Trial Center, Daejeon Korean Medicine Hospital of Daejeon University, Daejeon, Republic of Korea; 7 Research Center of Traditional Korean Medicine, College of Korean Medicine, Wonkwang University, Iksan, Republic of Korea; 8 Department of Il-won Integrated Medicine, Wonkwang University Korean Medicine Hospital, Iksan, Republic of Korea; 9 Korean Medicine Cognitive Disorder Research Center, College of Korean medicine, Wonkwang University, Iksan, Republic of Korea; University of Thessaly Faculty of Medicine: Panepistemio Thessalias Tmema Iatrikes, GREECE

## Abstract

**Objective:**

Despite the rapid increase in dementia and cognitive impairment incidence in Korea, research on integrative treatment for cognitive impairment using Korean medicine (KM) is still in its infancy. Thus, prospective studies with systematic data collection are required. This study aims to systematically collect and explore data from patients with dementia and mild cognitive impairment (MCI) who visit KM institutions. The data collected will include the participants’ baseline characteristics, cognitive impairment severity, KM diagnosis and treatment status, as well as the factors influencing their choice of integrative medical treatment.

**Materials and methods:**

This registry study will be conducted from the time of registration in 2024 until December 31, 2029, at Wonkwang University Korean Medicine Hospital, Wonkwang University Jangheung Integrated Medical Hospital, and Daejeon Korean Medicine Hospital of Daejeon University. Approximately 300 participants will be enrolled and visit the hospital annually for data collection. The collected data will include sociodemographic characteristics, laboratory tests, medical device inspections, long-term care information, and various questionnaires related to dementia and cognitive impairment. No predefined interventions or restrictions on treatment will be imposed. Standard and KM treatments for cognitive impairment, including combination therapies, are permitted. As a registry study, the purpose is to investigate the participants’ characteristics as outlined in the study objectives, including severity, KM diagnosis and interventions, and clinical outcomes. This epidemiological study is designed to include additional statistical analyses in response to research questions that emerge over time.

**Discussion:**

This study represents a pioneering effort in the KM field establishing the first registry of its kind focusing on dementia and MCI. This study aims to identify the characteristics of patients with dementia and MCI who visit KM institutions, explore the factors influencing KM treatment, and observe clinical outcomes according to KM pattern identification, providing evidence based on real-world data.

## Introduction

Global aging has accelerated over the past few decades. In 2022, the global population aged 65 years and older will reach 770 million, tripling the 1980 figure of 258 million. The older adult population continues to grow steadily, with projections of 994 million people by 2030 and 1.6 billion by 2050 [1]. This demographic shift reflects a significant risk for cognitive impairment, resulting in a rapid increase in the number of patients with dementia and mild cognitive impairment (MCI) worldwide [2]. The global prevalence of dementia is estimated to be approximately 55 million, with projections indicating it will increase to 139 million by 2050. Moreover, while 80% of individuals have expressed concerns regarding dementia, 25% believe nothing can be done to prevent it [3], indicating substantial apprehension regarding dementia onset. Dementia represents a severe loss of cognitive function that impairs social or occupational functioning [4], whereas MCI is considered an intermediate stage between normal aging and dementia [5]. Alzheimer’s disease accounts for 60–80% of dementia cases [2,6], followed by vascular dementia at 15–30% [7]. As dementia progresses, the need for caregiving and decision-making assistance increases, with patients ultimately requiring full-time care [8]. Consequently, dementia affects not only the individuals themselves but also their families and caregivers, imposing significant burdens on medical and care services and leading to substantial social costs. The global cost of dementia was estimated at $1 trillion in 2010 [9,10] and is projected to increase by approximately 85% by 2030, reflecting the largest economic burden of any single disease [3].

Despite the high socio-economic burden of dementia and cognitive impairment, the development of related treatments has been slow. Vascular cognitive impairment and dementia (VCID) require treatment for cerebrovascular issues in conjunction with dementia; however, no drugs have been approved for the specific treatment of VCID [11]. Moreover, the two drugs most utilized in current treatments are cholinesterase inhibitors (e.g., donepezil hydrochloride, cabalatin, and galantamine) and excitatory amino acid receptor antagonists (memantine) [12]. However, these options are limited in scope. In addition, monoclonal antibodies (mAbs) have recently received accelerated approval from the United States Food and Drug Administration to initiate treatment in patients with early Alzheimer’s disease with proven β-amyloid pathology (Aβ); however, long-term observation of brain volume loss and safety is required [13]. South Korea, which is in East Asia, is among the countries experiencing the most rapid aging worldwide. This demographic shift presents a significant healthcare challenge, as cognitive impairment prevention and management are becoming increasingly important. According to the Korean Statistical Information Service [14], in 2022, those aged 65 years and older accounted for over 18% of the total population, and this figure is projected to increase to 25.5% by 2030 and 43.8% by 2060. In contrast to the United Kingdom, which transitioned gradually from an aging society to a super-aged society over approximately 50 years, or the United States, which took approximately 15 years, Korea is expected to enter the super-aged society stage within a mere 7 years. Considering this accelerated aging, the National Institute of Dementia Annual Report 2022 [15] estimates that Korea has approximately 960,555 patients with dementia, with a prevalence of 7.3%. The projected cost of dementia management is expected to double every decade, reaching an estimated 78 trillion KRW by 2050.

The healthcare system in South Korea is structured on a dual framework, wherein traditional Korean medicine (KM) and Western medicine (WM) are officially recognized as equal. Consequently, patients have the option to select KM or WM, or a combination of both, for treatment based on their condition and symptoms. Furthermore, the National Health Insurance Service provides coverage for KM treatments, ensuring relatively high accessibility. In order to facilitate patient convenience and effective treatment, integrative medicine is often provided as a form of collaborative care [16]. A study utilizing data from the Health Insurance Review and Assessment Service found that 1.8% of all patients with dementia opted for KM treatment [17]. Furthermore, a survey conducted in 2022 on KM use revealed that 6.8% of hospital admissions to KM facilities were for dementia-related purposes [18]. However, in contrast to China, where diverse integrative management strategies for dementia and cognitive impairment are actively pursued [19], research on integrative treatment for cognitive impairment using traditional medicine is still in its nascent stages in Korea. KM has long addressed the characteristic symptoms of dementia under categories such as “Chimae (癡呆)” for dementia, “Geonmang (健忘)” for forgetfulness, and “Heoro (虛勞)” for exhaustion [20], and several previous studies have explored the effects of traditional medicine management on dementia and cognitive impairment [21,22]. Nevertheless, despite the chronic nature of diseases such as dementia, which necessitate prolonged tracking, most studies have encountered limitations owing to the relatively short duration of the studies themselves. Another limitation of the existing research is the lack of consideration of participants’ baseline characteristics and the influence of traditional medicine patterns on treatment [23]. Consequently, the systematic collection of data to assess the status and efficacy of traditional medicine in treating cognitive impairment is increasingly necessary [24]. Furthermore, although randomized controlled trials (RCTs) are considered the gold standard in evidence-based research, the importance of real-world data and evidence has been increasingly emphasized [25]. Consequently, studies that accurately reflect the actual clinical setting environment of traditional medicine institutions and systematically accumulate and manage data over extended follow-up periods are urgently needed.

This study is planned as a structured patient registry research project designed to collect, store, retrieve, analyze, and disseminate information on individuals diagnosed with specific diseases or experiencing health-related events [26]. Patients diagnosed with dementia or MCI who visit KM institutions will be enrolled in the study. Participants’ baseline characteristics, cognitive impairment severity, traditional medicine patterns, factors influencing the choice of integrative medicine treatment, and other relevant data will be systematically collected and explored. The participants will be classified into three groups based on whether they are receiving KM treatment, WM treatment, or a combination of both. The factors influencing the prognosis of cognitive impairment with integrative medical treatment will be analyzed based on the characteristics, clinical outcomes, and safety profiles of each group.

## Materials and methods

The protocol was registered on the clinical research registration website called the Clinical Research Information Service (CRIS). The registration number is KCT0009477. The URL is as follows: https://cris.nih.go.kr/cris/search/detailSearch.do?seq=27329&search_page=L.

### Registry aims

1. To gain a deeper understanding of the characteristics of patients with dementia and MCI.
2. To evaluate the clinical outcomes and safety of KM and standard treatment for dementia and MCI.
3. To investigate the factors that influence KM treatment of dementia and MCI.
4. To establish a registry of patients with dementia and MCI to facilitate long-term follow-up of the therapeutic effects of integrative medicine.

### Design and setting

**Fig 1:**
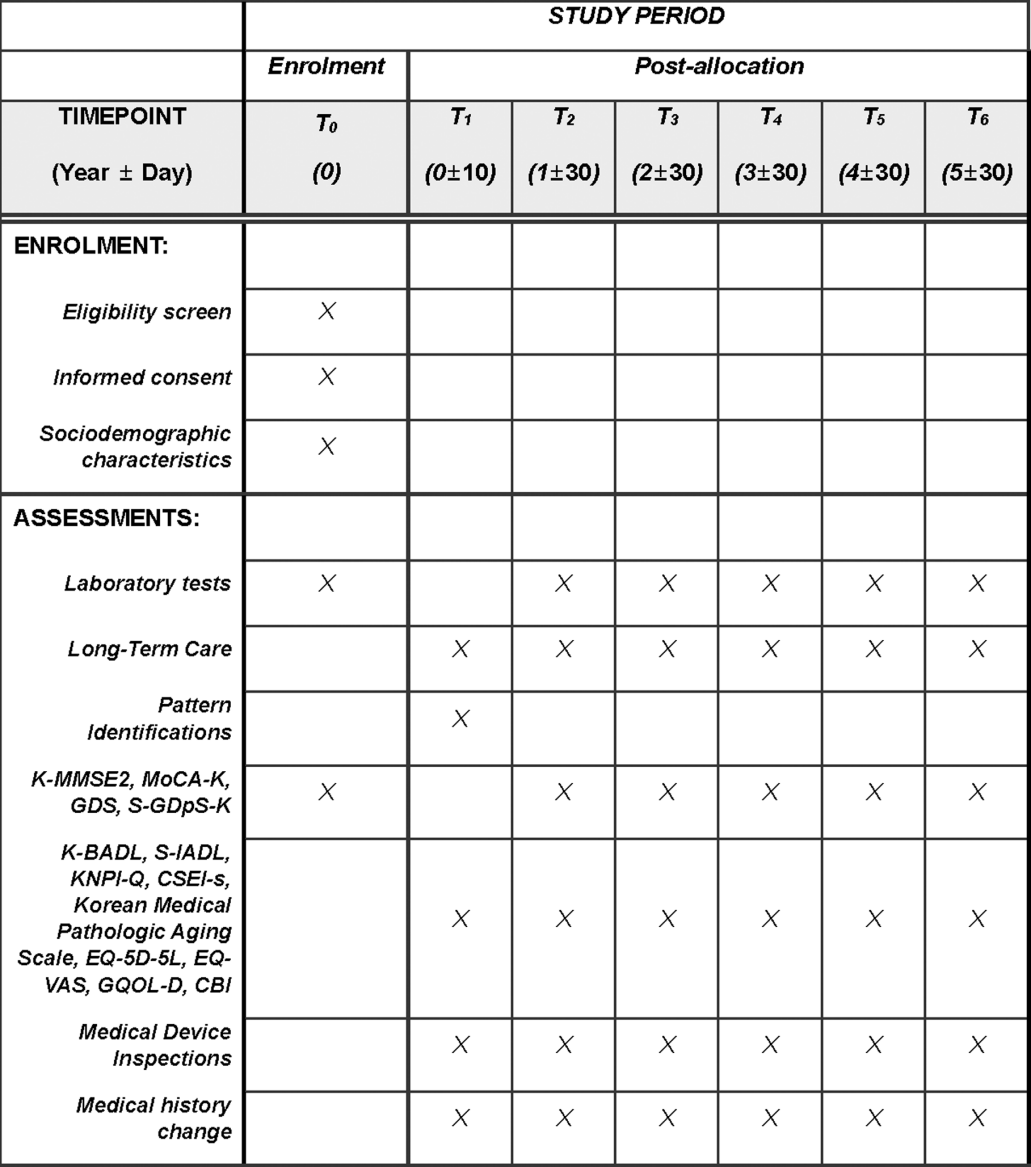
SPIRIT schedule of enrolment and assessments.

This is a prospective multi-center observational registry study of patients with dementia and MCI who visited academic hospitals that provide KM. Considering the number of patients with the respective diseases and accessibility over the past three years, three participating institutions were selected: Wonkwang University Korean Medicine Hospital, Wonkwang University Jangheung Integrated Medical Hospital, and Daejeon Korean Medicine Hospital of Daejeon University. A list of the participating institutions can be found in S2 Study Protocol v1.2. Participants enrolled in the registry will undergo annual follow-up visits to the hospital between the time of registration and December 31, 2029. Fig 1 presents a detailed schedule. The Short form Geriatric Depression Scale (S-GDpS-K), Global Deterioration Scale (GDS), Korean version of the Montreal Cognitive Assessment (MoCA-K), Korean-Mini Mental State Examination 2nd Edition: Standard Version (K-MMSE-2: SV) will be used to assess the primary outcome. Secondary outcome variables will be assessed using a sociodemographic survey, laboratory tests, a KM questionnaire, and measures of quality of life, patient health status, and caregiver burden. The study will not prescribe any predefined interventions; participants will receive treatment at their own or healthcare professionals’ discretion.

#### Study population and recruitment.

The study population comprises patients diagnosed with dementia and MCI who visit each participating institution and meet the inclusion criteria. Each participating institution plans to promote the study by posting recruitment posters on hospital websites or offline bulletin boards. Upon patient visits, screening evaluations will be conducted to assess whether they meet the inclusion and exclusion criteria. Patients who agree to participate will be provided with detailed information about the study and all safety-related matters before they are asked to provide informed consent. Furthermore, because this study is a government-funded project of the National Development Institute of Korean Medicine (NIKOM), data may be anonymized and linked to secondary data sources, such as national health insurance claims data, for future use. Accordingly, separate consent will be obtained for granting access to third parties and use in secondary research. The electronic Case Report Form (e-CRF) is established by the NIKOM and the information is managed by the government. Such registries are being conducted simultaneously for other diseases, and the government wants to continue research and manage data. We will conduct an observational study for 7 years with funding and then continue to receive funding to build the registry and conduct related research.

#### Inclusion criteria.

1. Adults aged 55–85 years.
2. Individuals diagnosed with a major neurocognitive disorder caused by Alzheimer disease or vascular disease, or a mild neurocognitive disorder based on the criteria described in the *Diagnostic and Statistical Manual of Mental Disorders, 5th Edition* (DSM-5).
3. Individuals who voluntarily agree to participate or whose legal representatives have provided signed informed consent.

#### Exclusion criteria.

1. Patients with dementia caused by conditions other than Alzheimer disease or vascular dementia (e.g., degenerative brain diseases such as Parkinson disease, Huntington disease, frontotemporal disorder, and Creutzfeldt–Jakob disease).
2. Individuals with systemic conditions that may cause dementia (e.g., hypothyroidism, vitamin B12 or folic acid deficiency, niacin deficiency, hyperkalemia, neurosyphilis, and human immunodeficiency virus disease).
3. Individuals with a history of being diagnosed with a psychotic disorder or substance-related disorder based on DSM-5 criteria (e.g., schizophrenia, delusional disorder, bipolar disorder, and alcohol or substance abuse disorder).
4. Individuals with a history of neurological disorders such as epilepsy, focal brain injury, and head trauma.
5. Individuals with gastrointestinal, endocrine, or cardiovascular diseases that cannot be controlled through dietary or drug therapy.
6. Individuals in a serious unstable medical condition (as determined by a physician based on laboratory tests, electrocardiogram results, chest X-rays, and vital signs).
7. Others whom the investigator deems ineligible to participate in the study.

#### Sample size.

The sample size was calculated as follows: Through discussions among the research team, we pre-selected 15 covariates that are expected to affect changes in MOCA-K, an important continuous variable for evaluating treatment progress. The 15 covariates are as follows: gender, age, smoking, alcohol consumption, weight, height, cognitive impairment severity, region of residence, duration of illness, income, education level, occupation, marital status, medication use, and family history. We aimed to conduct a multiple linear regression analysis to identify which covariates influence changes in MOCA-K for patients receiving Korean medicine treatment. It is generally known that multiple linear regression analysis requires approximately 20 samples for each independent variable, so the total sample size was estimated to be around 300. As this study is intended as an epidemiological investigation inherent to the nature of the registry, no predefined statistical hypotheses have been formulated. As new research questions emerge over time, additional statistical analysis plans will be devised. Consequently, each participating institution has estimated the number of eligible patients who could reasonably be registered to calculate the final sample size. The average number of patients with dementia and MCI across the three institutions is 121 per year. With an estimated registration rate of approximately 50%, the objective is to enroll 60 new participants annually, with the goal of obtaining a total of 300 new registrations in total over five years.

### Data management

#### Data analysis.

The analysis plan for the research questions that arise over time is as follows. Descriptive analyses will be employed to demonstrate the baseline data and disease-related variables. Nominal variables will be presented as frequencies and ratios, whereas continuous variables will be presented as means and standard deviations. Baseline data will be subjected to statistical analysis to identify associations between the data and several variables, including treatment characteristics, treatment effects, factors influencing KM treatment, health outcome variables, and prognosis. Changes in continuous variables in all patients or specific subgroups will be tested using either paired t-tests or Wilcoxon signed-rank tests, depending on the normality test results. Changes in the nominal variables will be tested using McNemar’s test. Correlations between continuous variables will be analyzed using Pearson (or Spearman) correlation analysis. Differences in continuous variables between subgroups will be analyzed using independent t-tests (or Wilcoxon rank-sum tests, depending on the normality test results). Differences in nominal variables will be analyzed using chi-squared tests (or Fisher’s exact test, depending on the normality test results). Multiple regression analysis will be used to test the effects of the covariates on a particular continuous dependent variable, and multinomial logistic regression analysis will be used to test the effects of the covariates on the occurrence of binominal indicators. Survival analysis will be performed to test the effects of the covariates on the occurrence of a particular binomial variable over time. The proportion of missing data that can be included in the analysis is limited to 10%. The missing data will be imputed using one of several strategies, including unconditional or conditional mean imputation and expectation maximization.

#### Data collection.

This study is a pure research project with no feedback to participating hospitals and aims to collect basic patient sociodemographic characteristics, survey information, and clinical practice data. All documents pertaining to registry participants will be recorded using identification codes rather than names and stored on computers as encrypted files with access restricted to approved researchers only. Trained trial staff and research coordinators will be responsible for collecting baseline and follow-up data. All data will be stored in an e-CRF using an electronic data capture system called MyTrial, which is managed by the Korea Institute of Oriental Medicine. The online database permits information to be modified and updated as required and real-time exportation for the purposes of quality management and statistical analysis purposes. Once permission has been granted for the data to be used for public purposes, it will be accessible via the online database.

#### Data quality control and queue maintenance.

Data completeness and reliability will be evaluated by independent monitoring personnel separate from the researchers and sponsors. The monitoring personnel will be responsible for ensuring compliance with the observational study protocol, endorsing modifications of the registry protocol, appropriate and accurate data collection, and confirmation of participants’ informed consent. If data completeness and reliability are compromised, face-to-face or telephone interviews will be conducted with the participants to facilitate the necessary corrections. Furthermore, a random sample of less than 10% from each institution will be selected up to five times during the study period for comparison between the data entered in the e-CRF and the paper case report forms maintained at each institution. If the input errors exceed a predetermined acceptable level, the institution will compare all case records and input data. To minimize dropout rates, materials related to health and cognitive disorders, including lifestyle information, will be distributed at least once per year. Additionally, the study coordinator will contact the participants to monitor their health management status.

### Measures

Collect sociodemographic characteristics, lab and medical device tests, long-term care information, and various questionnaires from participants. Trained trial staff and research coordinators will be responsible for collecting sociodemographic characteristics, lab and medical device tests, long-term care information. Among various questionnaires, Pattern Identification Tool for Cognitive Disorder (PIC-C), Mini-Mental State Examination (MMSE), Montreal Cognitive Assessment (MoCA), GDS, Geriatric Quality of Life-Dementia (GQOL) are assessed by trained medical staff. In addition, the Barthel Index, Seoul-Instrumental Activities of Daily Living (S-IADL), Brief Questionnaire form of Neuropsychiatric Inventory (NPI-Q), and Caregiver Burden Inventory (CBI) will be assessed by the caregiver. And the S-GDpS-K, Core Seven-Emotions Inventory Short Form (CSEI-s), Korean Medical Pathologic Aging Scale, EuroQol 5 Dimensions 5 Levels and EuroQol Visual Analog Scale (EQ-5D and EQ-VAS) will be assessed by the participant with the help of the caregiver. Fig 1 presents a detailed schedule.

#### Sociodemographic characteristics.

Information on the sociodemographic characteristics and medical history of the participants and their caregivers will be collected.

#### Laboratory tests and medical device inspections.

Data will be collected from participants regarding hematology tests, blood chemistry tests, urinalysis, Vital signs, and medical device inspections (electrocardiogram, chest X-ray, heart rate variability, quantitative electroencephalograph, and functional near-infrared spectroscopy)

#### Long-term care.

Information will be collected on participants’ long-term care insurance grade, nursing home admission, and long-term care services currently in use.

#### Questionnaires.

**Pattern identification tool for cognitive disorder:** The PIC-C [20,27] comprises 22 items across 10 categories and classifies cognitive impairment into four patterns: Qi Deficiency, Yin Deficiency, Dampness, and Heat Syndrome. In addition to determining the primary pattern, it quantitatively assesses the tendencies of other patterns. Consequently, it can be utilized for evaluating the selection of primary prescriptions for herbal medicine and acupuncture treatment, adjusting herbal dosages, selecting adjunctive acupuncture treatments, and assessing treatment progress.

**Barthel index:** Various versions of the Barthel Index are available. Originally, the total score ranged from 0 to 100 points; however, this study used the version revised by Collin et al. [28], which was translated into Korean as the Korean version of Barthel Activities of Daily Living Index (K-BADL) [29]]. The K-BADL assesses the ability to perform basic activities of daily living. The scale is based on a total score of 20 points, with 11–15 points indicating moderate disability and ≤ 10 points indicating severe disability.

**Seoul-instrumental activities of daily living:** The S-IADL [30] was developed and standardized using items suitable for Korea’s cultural characteristics. It comprises 15 items rated on a scale of 0–3 points, with a total score ranging from 0 to 45 points. Higher scores indicate greater disability in daily life. This tool evaluates instrumental activities of daily living by dividing them into current and potential abilities. It has been demonstrated that a cut-off score of 7.5 can be used to differentiate between normal individuals and those with dementia. An S-IADL score of 8 points or above indicates the likely presence of dementia.

**Brief questionnaire form of neuropsychiatric inventory:** The NPI-Q [31] is a streamlined version of the original NPI [32] designed for straightforward use in clinical settings. The instrument evaluates the severity of 12 common neuropsychiatric symptoms observed in patients with dementia and their caregivers. It assesses both the severity of neuropsychiatric symptoms (rated 1–3) and caregiver distress caused by these symptoms (rated 0–5). Higher scores indicate greater severity. The Korean version of the NIP-Q (KNPI-Q) [33] will be used in this study.

**Mini-mental state examination 2nd edition: Standard version:** The MMSE [34] is the most widely used test for screening cognitive impairment in older adult populations. To provide greater sensitivity and adaptability to each country’s culture, the MMSE-2 was developed, which consists of three versions: Brief Version (BV), Standard Version (SV), and Expanded Version (EV). As with the original MMSE, the K-MMSE-2:SV has a total score of 30 points. It assesses orientation to time (5 points), orientation to place (5 points), registration (3 points), attention and calculation (5 points), recall (3 points), language (8 points), and drawing (1 point). A total score of 23 points is typically considered the cutoff point for cognitive impairment.

**Montreal cognitive assessment:** The MoCA [35], which has been translated into Korean [36], will be used. The MoCA-K is a questionnaire developed to screen for MCI in individuals with normal findings on the MMSE test. Total scores equal 30 points, and a score of 23 or higher is considered normal.

**Global deterioration scale:** The Korean version of the GDS will be used in this study [37,38]. The GDS is generally used to quantify the extent of dementia progression. GDS 1 indicates a clinically normal individual without cognitive impairment, GDS 2 indicates subjective memory impairment, and GDS 3 indicates MCI and some with mild dementia. From GDS 4 onwards, a definitive diagnosis of dementia can be made, with GDS 4 representing mild dementia, GDS 5 representing moderate dementia, and GDS 6 and 7 representing severe dementia.

**Short form geriatric depression scale:** This study will use the Korean version of the S-GDpS-K [39], which has been developed by shortening the original 30-item GDpS [40] to 15 items. This self-report questionnaire allows for simple “yes” or “no” responses. A score of 8 or higher indicates a depressive state.

**Core seven-emotions inventory short form:** The CSEI-s [41] consists of 28 items across 7 categories, with each item measured on a 5-point Likert scale. This scale uses t-scores (mean 50, standard deviation, 10) as reference points. For emotions other than happiness, including anger, thoughtfulness, sadness, fear, anxiety, and surprise, higher scores indicate a higher risk level. Regarding the specific cutoff points: t-scores ranging from 55 to 60 are considered cautionary, scores from 61 to 65 are considered risky, and scores above 66 are classified as high risk. In contrast, for happiness, lower scores indicate a higher likelihood, with t-scores ranging from 40 to 45 considered slightly likely, 35–39 considered likely, and scores below 34 classified as highly likely.

**Korean medical pathologic aging scale:** The Korean Medical Pathologic Aging Scale [20] comprises 9 items related to the sensory and physical symptoms of aging. Each item is measured on a 3-point Likert scale, with 0 points indicating “none,” 1 point indicating “sometimes present,” and 2 points indicating “frequently present.” Lower scores signify a better health status in relation to aging.

**EuroQol 5 dimensions 5 levels and EuroQol visual analog scale:** The EQ-5D [42,43] assesses health-related quality of life and comprises five dimensions: mobility, self-care, usual activities, pain/discomfort, and anxiety/depression. Each dimension is evaluated on a five-point scale, with 1 indicating the best state and 5 indicating the worst state. The EQ-VAS is a visual analog scale comprising a vertical line of 100 mm. The EQ-VAS represents the respondent’s self-rated health status on a scale of 0–100, on which 100 represents the best imaginable health state and 0 represents the worst imaginable health state. In general, rating scales are recommended as supplementary tools to other instruments. The EQ-VAS was also included as a supplementary component to the standard EQ-5D questionnaire.

**Geriatric quality of life-dementia:** The GQOL scale [44] is used to assess the comprehensive quality of life of older adults and patients with dementia. Subsequently, the Geriatric Quality of Life-Dementia scale (GQOL-D) [45] was developed to reflect the quality-of-life domains of particular importance to patients with dementia. The GQOL-D comprises 13 items that assess various quality-of-life domains, including physical health, psychological health, independence level, social relations, environment, and religion. It also includes one item for overall health and another item for overall life satisfaction. Each item is measured on a 4-point Likert scale.

**Caregiver burden inventory:** The CBI [46] assesses the burden experienced by caregivers of patients with dementia. The scale has 24 items in total, divided into five domains: time-dependent burden, developmental burden, physical burden, social burden, and emotional burden. One advantage of the CBI is that it allows for the simultaneous measurement of both objective and subjective burden.

#### Adverse events.

This registry study is designed without any interventions included and has a low expectation of risks or adverse events (AEs). All medical histories collected during the follow-up visits after the initial visit will be documented as medical histories, and only recorded as serious AEs if the participant dies. Additionally, cumulative mortality cases from all participating institutions will be reported collectively during periodic reviews. Clinical research indemnity insurance has been obtained to mitigate potential harm from trial participation.

### Ethical considerations

This study was approved by the Institutional Review Board of Wonkwang University Korean Medicine Hospital (February 16, 2024; IRB 2023-14), Institutional Review Board of Wonkwang University Jangheung Integrative Medical Hospital (March 14, 2024; WKUJIM-202403–003), and Institutional Review Board of Daejeon Korean Medicine Hospital of Daejeon University (February 29, 2024; DJDSKH-23-BM-14).

Before obtaining consent from prospective participants, the researcher will thoroughly explain the research protocol and consent form, both of which have received Institutional Review Board approval. Written informed consent will be obtained in accordance with ethical principles based on the Declaration of Helsinki. Once written consent has been obtained from participants, they will be provided with a copy of the consent form. To ensure the participants’ confidentiality, they will each be assigned a unique identification number. Records pertinent to the study will be retained for three years following the conclusion of the study, after which they will be destroyed in accordance with the provisions of the Bioethics and Safety Act. However, the retention period can be extended if deemed necessary by the sponsor. Information pertaining to research conducted to provide data to third parties for secondary research utilization will be retained, managed, and provided for disposal upon request or for up to 10 years following the completion of the primary study. Individuals diagnosed with major neurocognitive disorders are classified as vulnerable, and both the participant and their legal representative must provide consent. Those diagnosed with MCI are deemed capable of providing their own consent. Should the researcher identify a change in the participant’s medical condition subsequent to the initial provision of consent, and should the participant be deemed vulnerable, additional consent will be obtained from both the participant and their legal representative. The researcher will assess the legal representative’s capacity to consent through outpatient visits. If the representative’s consent capacity is impaired, the participant will not be registered or consent will be obtained from another legal representative.

### Status and timeline of the registry

Participant enrolment for the registry will begin in August 26, 2024

## Discussion

This registry study will conduct long-term observation of patients with dementia and MCI who attend university-affiliated KM hospitals. In addition, the registry established in this study will be used for long-term epidemiological research by linking it with health insurance and retrospective hospital data.

Although RCTs have been published on the effectiveness and safety of East Asian traditional medicine for dementia [47,48], recent studies have emphasized the need for real-world data and evidence [25]. The objective of this study is to expand the current evidence base by systematically collecting real-world data in conjunction with existing RCT results. While retrospective cohort studies using health insurance data offer advantages, such as large sample sizes and long follow-up periods [49,50], they also have limitations, including the data not being collected specifically for research purposes and other limitations inherent in the data itself [51]]. This study aims to overcome the limitations of extant research by prospectively collecting multiple outcome variables and combining them with retrospective data to provide new insights. This study represents the inaugural registry for dementia and MCI in the field of KM and is the first longitudinal study to apply Korean medical questionnaires such as the PIT-C, CSEI-s, and the Korean Medical Pathologic Aging Scale. This study will explore the factors that influence KM treatment to provide a clearer understanding of the characteristics of patients with dementia and MCI who visit KM institutions. Furthermore, through long-term observation of patients based on the identification of their KM patterns, evidence will be accumulated for the development of personalized treatments for dementia and MCI.

### Limitations

The study has a relatively short follow-up period of up to 5 years, and as a registry study, it lacks random allocation, which may introduce various confounding factors [52,53]. Since there was no predetermined intervention, clinical interventions may vary depending on the clinician. The study will be conducted at three university-affiliated hospitals, selected to represent the diversity of healthcare systems in Korea. The hospitals are located in a 1.5 million metropolitan area, a 250,000 urban area, and a 40,000 rural area. However, the limited number of participating hospitals and the study’s regional scope may limit its representativeness. Finally, this study will be conducted in university-affiliated KM hospitals and may not be representative of primary care institutions.

### Dissemination

The findings of this research will be presented in settings such as academic conferences and international seminars, as well as published in peer-reviewed journals. Furthermore, the findings will be used in developing clinical practice guidelines for treating cognitive impairment. Additionally, the findings of this research will be reported annually, and a dedicated website will be created to provide further information.

## Supporting information

S1 File SPIRIT 2013 checklist: Recommended items to address in a clinical trial protocol and related documents. (DOCX)

S2 File Study protocol for IRB v1.2_English (Effective Date: 2024. 01. 22). (DOCX)

S3 File Study protocol for IRB v1.2_Korean (Effective Date: 2024. 01. 22). (DOCX)

## Abbreviations:

KM: Korean medicine
MCI: mild cognitive impairment
RCTs: randomized controlled trials
VCID: vascular cognitive impairment and dementia
