# Supplementary material for: Korean medicine registry for cognitive disorder: A protocol for prospective observational multi-center study
Source: PLoS One. 2025 May 15;20(5):e0323170. doi: 10.1371/journal.pone.0323170 (PMC12080776; doi:10.1371/journal.pone.0323170)
Supplement: S2 File — (DOCX) [file pone.0323170.s002.docx]

Clinical Study Protocol

**Korean Medicine Registry for Cognitive Disorders**

| Protocol No. | WKH-CIR-2023 |
| --- | --- |
| Version | 1.2  (Effective Date: 2024. 01. 22.) |
| Study Center | Wonkwang University Korean Medicine Hospital |
| Collaborating Centers | Wonkwang University Jangheung Integrative Medical Hospital  (Principal Investigator: Hyung-Won Kang)  Daejeon University Daejeon Korean Medicine Hospital  (Principal investigator: In-Chul Jung) |
| Sponsor | Not applicable (Investigator-initiated clinical trial) |
| Principle Investigator | Jung-Tae Lim |

| ***CONFIDENTIAL*** |
| --- |
| All information related to this protocol shall be kept confidential and may not be disclosed without prior written consent. |

**▣ Protocol Revision History**

| **No** | **Version No.** | **Version Date** | **Key Details** |
| --- | --- | --- | --- |
| 1 | 1.0 | 2023. 10. 26. | Initial draft of the clinical study protocol. |
| 2 | 1.1 | 2023. 10. 30. | Revised to Version 1.1 to incorporate feedback from other centers before submission to the Institutional Review Board (IRB) of Wonkwang University Korean Medicine Hospital. |
| 3 | 1.2 | 2024. 01. 22. | Revised to Version 1.2 to address the review comments from the IRB of Wonkwang University Korean Medicine Hospital and the IRB of Daejeon University Daejeon Korean Medicine Hospital. |

**Protocol Agreement**

Korean Medicine Registry for Cognitive Disorders

Protocol Number: WKH-CIR-2023

Version: 1.2

Date Prepared: 2024.01.22.

- I have read the protocol and hereby agree to conduct this study in accordance with all applicable regulations and guidelines.
- I hereby agree to hold in strict confidence all information developed or obtained in relation to this protocol.

|  |  |  |
| --- | --- | --- |
| **Principal Investigator**  Jung-Tae Lim  Wonkwang University Korean Medicine Hospital |  | **Date** |

**Protocol Agreement**

Korean Medicine Registry for Cognitive Disorders

Protocol Number: WKH-CIR-2023

Version: 1.2

Date Prepared: 2024.01.22.

- I have read the protocol and hereby agree to conduct this study in accordance with all applicable regulations and guidelines.
- I hereby agree to hold in strict confidence all information developed or obtained in relation to this protocol.

|  |  |  |
| --- | --- | --- |
| **Principal Investigator**  Hyung-Won Kang  Wonkwang University Jangheung Integrative Medical Hospital  (Principal investigator for the entire research project) |  | **Date** |

**Protocol Agreement**

Korean Medicine Registry for Cognitive Disorders

Protocol Number: WKH-CIR-2023

Version: 1.2

Date Prepared: 2024.01.22.

- I have read the protocol and hereby agree to conduct this study in accordance with all applicable regulations and guidelines.
- I hereby agree to hold in strict confidence all information developed or obtained in relation to this protocol.

|  |  |  |
| --- | --- | --- |
| **Principal Investigator**  In-Chul Jung  Daejeon University Daejeon Korean Medicine Hospital |  | **Date** |

**Table of Contents**

**1. Title** **6**

**2. Background** **6**

**3. Objectives 10**

**4. Name and Address of Study Centers** **11**

**5. Principal Investigator at Each Center** **12**

**6. Study Period** **12**

**7. Study Subjects** **13**

**8. Sample Size and Rationale** **13**

**9. Methods and Procedures** **14**

**10. Data Collection and Management** **25**

**11. Data Analysis** **25**

**12. Subject Compensation** **26**

**13. Protection of Privacy and Handling of Personal Information** **26**

**14. Expected Risks and Benefits to the Subjects** **29**

**15. Safety Protection Measures and Indemnification** **30**

**16. Data Storage** **31**

**17. References** **32**

**1. Clinical Study Title**

Korean title: 인지장애 한의 레지스트리

English title: Korean Medicine Registry for Cognitive Disorders

**2. Background**

□ Korea is expected to enter a super-aging society by 2025, with the elderly projected to account for 43.9% of the total population by 2060.

○ A report by Statistics Korea indicates that Korea will achieve super-aging status by 2025, only seven years after becoming an “aged society” in 2018. The elderly are expected to account for 43.9% of the total population by 2060.

○ Due to the decline in the productive-age population (aged 15-64 years), the old-age dependency ratio—measuring the population aged 65 years and older per 100 population— is projected to increase from 21.7 in 2020 to 51.0 in 2036, ultimately reaching 91.4 in 2060.
- Government support, including basic pension, for the elderly aged 75 and older increased from 12.834 trillion won in 2010 to 28.96 trillion in 2016.

[Increasing trend in elderly population aged 65 and older]


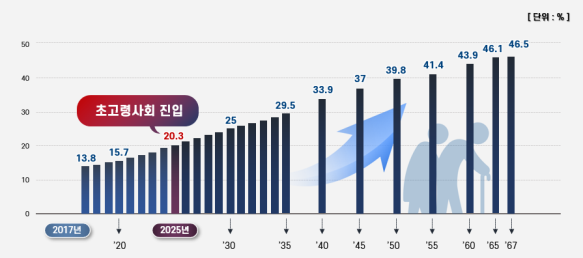


Translation of Korean words in the figure

Unit

Entry into super-aged society

2017 2025

□ Medical expenses for the elderly exceeded 40% of the total medical expenses in 2018, and they are expected to reach 60 trillion won for those aged 65 and older in 2025.

□ There is a growing need for aging-related research to promote healthy aging and enhance the quality of life (QoL) in a super-aging society.

○ Therapeutics for aging-associated diseases have low efficacy, while there is an increasing demand for “healthy aging,”* which focuses on aging in a healthy state through early prevention and management.
* The World Health Organization (WHO) has announced the “Decade of Healthy Aging (2021-2030)” plan, which aims to promote healthy aging by reducing disease incidence, maintaining high cognitive and physical functioning, and encouraging active participation in social activities. This is in contrast to “usual aging,” which is accompanied by the normal aging process and aging-associated diseases.

○ With the global population aging, aging-related research is emerging as a new growth industry.
- The global anti-aging and service market was valued at $62.5 billion in 2017 and was anticipated to grow at an annual average of 6.5% to reach $88.6 billion (109 trillion won) in 2022.

□ In 2019, there were 788,000 individuals with dementia among the Korean population aged 65 years and older.

○ According to the Ministry of Health and Welfare Central Dementia Center, this number is gradually increasing. As a result, the cost of dementia care has doubled every 10 years, and the social cost associated with dementia is expected to reach approximately 78 trillion won by 2050. In response, the Korean government announced the “National Responsibility for Dementia Care” in 2017 to focus on dementia prevention and effective treatment.

○ Recently, global pharmaceutical companies have abandoned their efforts to develop therapeutics for dementia, leading to increased interest in treating cognitive impairment that precedes dementia. Studies have reported that 10-41% of patients diagnosed with mild cognitive impairment (MCI) will develop dementia within a year, although results vary among researchers. These findings suggest the need for proactive management and treatment starting from per-dementia cognitive impairment.

○ The 2016 Nationwide Survey on Dementia Epidemiology in Korea found that the risk of dementia was approximately 4.6 times higher among individuals with depression than among those without depression. Depression is considered a pre-symptom or symptom of dementia, and a strong association between depression and dementia in the elderly has been reported. Early diagnosis of dementia relies on recognizing MCI and senile depression as important variables, as research has shown that these variables also affect prognosis.

□ Although the effectiveness of Korean Medicine (KM) for early dementia has been demonstrated, KM treatment has not been widely embraced by patients with dementia.

○ Dementia and cognitive disorders, common neurological disorders in the elderly, require alternatives to drug therapy to prevent the gradual deterioration of cognitive function and address behavioral and psychological symptoms, as well as provide emotional support.

○ KM treatment has shown efficacy in alleviating symptoms of senile dementia and cognitive disorders by improving cognitive function, supporting patients with behavioral and psychological symptoms, and providing family care. Therefore, having frontline KM doctors treat and manage dementia can offer various choices to the public and provide more benefits to patients with cognitive impairment and high-risk groups.

□ There is a need to promote KM treatment for patients with dementia.

○ In 2011, the Ministry of Health and Welfare implemented the “Dementia Examination Program” for early detection of dementia. as stipulated in Article 11-1 of the Dementia Management Act. However, KM is excluded from the list of medical institutions that can provide treatment after a dementia diagnosis.

○ This exclusion has persisted for nearly a decade, despite the establishment of a dementia health center program that grants the right to diagnose dementia but not to treat it. There is a need for stronger guarantees for designated contract hospitals and clinics following health center examinations.

○ The lack of evidence for institutional entry has become a critical issue. There is an urgent need to generate evidence reflecting clinical settings to expand coverage and reimbursement for herbal medicine, making KM technology more accessible to patients with cognitive disorders.

○ Establishing a registry for cognitive disorders such as MCI and dementia, as well as late-life depression, which is highly associated with both depression and dementia in the elderly, can enable comparative analysis with other existing databases. This analysis can help estimate the morbidity rate for progressing from MCI or depression to dementia based on the KM treatment course, as well as from dementia to mortality. Therefore, it is necessary to establish a cognitive disorder registry that can prioritize the creation of evidence for KM treatment.

○ Article 2 (Definitions), section 2: Despite having legal guarantees for the right to diagnosis, the right to treatment has not been guaranteed. Only KM neuropsychiatrists can issue dementia special rating reports, and KM doctors are excluded from the list of doctors affiliated with dementia relief centers.

□ KM offers various prevention and management measures for healthy aging.

○ Acupuncture, moxibustion, and herbal medicine are used in KM to regulate the fundamental energy of the human body, which have been reported to be effective in preventing and treating aging-associated diseases.
- Aging causes changes in biorhythms, including hormone secretion, sleep patterns, body temperature maintenance, and blood pressure, which can lead to aging-associated diseases and hinder healthy aging.
- The Shinhyeongmun section of Donguibogam presents various measures for healthy aging based on living according to nature and one’s own biorhythms, such as the “four seasons,” “navel method,” “warming the navel,” “navel moxibustion,” and “fetal breath.”

□ Local government-centered KM dementia program at health centers for the elderly with cognitive disorders.

○ In 2016, the Seoul Korean Medicine Association secured a city budget of 500 million won to conduct a pilot program involving 146 KM clinics. The program, which ran for 4 and 8 weeks, received a satisfaction score of 9.02 out of 10 points. This program is seen as providing evidence for expanding the scope of KM, in line with the National Responsibility for Dementia Care policy. Other local government-centered KM dementia programs at health centers include those in Busan and Gyeonggi Province.

○ The Moon Jae-in government’s five-year plan for state administration included policies on mental health. Accordingly, there was a strengthened linkage to KM dementia treatment guidelines in the national strategies, which aimed to enhance the mental health promotion system and implement the National Responsibility for Dementia Care policy (Table 1).

| **Specific implementation system** | **Details** |
| --- | --- |
| Strengthening the mental health promotion system | - Restructuring the mental health-related service delivery system by hiring more specialists and improving their working conditions.  - Expanding prevention of suicide and fostering a culture of respect for life, aiming to increase the utilization of mental health services from 15% to 20%. |
| National Responsibility for Dementia Care | - Expansion of 252 dementia relief centers nationwide and establishment of dementia relief hospitals starting in 2017.  - Reduction of out-of-pocket expenses for patients with severe dementia and expanded insurance coverage for high-cost diagnostic tests starting in 2018.  - Expanded reduction of out-of-pocket expenses for long-term care recipients with dementia. |

□ Status of research on geriatric cognitive disorders

(1) Status of cohorts and hospital cohorts (CREDOS) related to cognitive disorders, including senile dementia, in Western medicine (WM)

○ Cohorts for senile dementia research have been ongoing since 2006. According to data from the Central Dementia Center, the number of patients with dementia in Korea is estimated to be approximately 840,000, with projections of approximately 1.27 million in 2030 and 2.71 million in 2050.

○ Data from the Health Insurance Review and Assessment Service on the analysis of medical services for dementia diseases based on nationwide medical services over an 11-year period (2009-2019) indicate that the number of dementia patients and cases increased by 3.6 and 5.5 times, respectively. Medical costs increased by 7.3 times, representing a larger increase compared to the 3.4-fold increase in total medical costs.

○ Among dementia diseases, Alzheimer’s disease accounted for the highest medical care benefit cost in 2018, approximately 1.4 times higher than the next highest disease, cerebral infarction. In addition, the National Health Insurance Policy Research Institute has initiated the third project in the “establishment of long-term care elderly cohort.” This study aims to provide evidence for policy development to achieve “Healthy Aging Place” in the community.

(2) Status of KM treatment technologies and intellectual properties related to geriatric cognitive disorders

○ Clinical symptoms such as memory decline, cognitive dysfunction, emotional behavioral problems, and personality changes, which are seen in neurocognitive disorders, are addressed in KM under categories such as dementia, dementia disease, forgetfulness, madness, and emptiness.

○ The KM assessment tool for dementia cannot yet be used as a standardized diagnostic tool in clinical practice due to a lack of objectification and standardization. Lee et al. conducted a literature review and expert opinion survey to create a scale that assesses the clinical symptoms of cognitive disorders based on four patterns: Qi deficiency, Yin deficiency, HwaYul, and Dam-eum. They conducted a clinical trial to create the Pattern Identifications Tool for Cognitive Disorders (PIT-C) Ver. 2.1. Additionally, Lee et al. conducted a clinical study to confirm the reliability and validity of PIT-C Ver. 2.1.

(3) Status of KM research related to geriatric cognitive disorders

○ After applying acupuncture therapy to patients with MCI, changes in brain activities were observed using fMRI, along with improvements in clinical cognitive function markers. As a result, changes in activities in 20 brain regions were measured. The elderly patient groups showed significant improvements in the Clinical Dementia Rating (CDR) scale and Mini-Mental State Examination (MMSE) scores.

○ In a meta-analysis of five clinical studies involving 578 subjects on acupuncture therapy for amnestic MCI, the acupuncture therapy group showed greater clinical effects than the group treated with nimodipine, a cerebral vasodilator.

○ Herbal medicine is known to have fewer adverse events and excellent efficacy compared to drug therapies for MCI.

○ In a clinical study on Shenwu capsules for patients with amnestic MCI, the Shenwu-treated group showed an equivalent level of memory loss prevention effect as the donepezil-treated group for up to 48 weeks, with fewer gastrointestinal and neuropsychiatric adverse effects than the donepezil-treated group.

□ KM treatment has shown certain efficacy in alleviating symptoms of senile dementia and cognitive disorders by improving cognitive function, supporting patients with behavioral and psychological symptoms, and providing family care. Therefore, having frontline KM doctors treat and manage dementia can offer various choices to the public and provide more benefits to patients with cognitive impairment and high-risk groups. Nonetheless, there are no actual cases in KM to date where data regarding such dementia diseases were collected and analyzed to establish a database.

**3. Objectives**

□ Ultimate study objective

○ This study aims to establish a KM registry for the accumulation of data on KM diagnosis, treatment, management, and prevention for patients with cognitive disorders (MCI, Alzheimer’s disease, vascular dementia) and to collect data on factors that affect cognitive function.

**4. Name and Address of Study Centers**

**1) Sponsoring center and study centers**

**(1) Sponsoring center**

| **Name** | **Address** | **PI** | **Telephone** |
| --- | --- | --- | --- |
| Wonkwang University  Jangheung Integrative Medical Hospital (WKU JHIMH) | 121 Rohaseu-ro, Anyang-myeon, Jangheung, Jeollanam-do, South Korea | Hyung-Won Kang | 061-860-7777 |

**(2) Study centers**

| **Name** | **Address** | **PI** | **Telephone** |
| --- | --- | --- | --- |
| WKU JHIMH | 121 Rohaseu-ro, Anyang-myeon, Jangheung, Jeollanam-do, South Korea | Hyung-Won Kang | 061-860-7777 |
| Wonkwang University Korean Medicine Hospital (WKU KMH) | 895 Muwang-ro, Iksan, Jeollabuk-do, South Korea | Jung-Tae Lim | 063-850-6914 |
| Daejeon University Daejeon Korean Medicine Hospital (DJU DKMH) | 75 Daedeok-daero 176beon-gil, Seo-gu, Daejeon, South Korea | In-Chul Jung | 042-470-9129 |

**2) Statistics related to cognitive disorders from the sponsoring center and study centers**

**(1) JHIMH**

- In 2020, there were a total of 15,153 patients, including 9,598 patients aged ≥ 60 years (63.3% of all patients), and 19 patients with MCI or dementia (0.1% of all patients).

- In 2021, there were a total of 25,508 patients, including 8,264 patients aged ≥ 60 years (55.4% of all patients), and 17 patients with MCI or dementia (0.1% of all patients).

- In 2022, there were a total of 27,623 patients, including 12,834 patients aged ≥ 60 years (56.7% of all patients), and 22 patients with MCI or dementia (0.2% of all patients).

- Overall, there was an increasing trend in the number of patients aged ≥ 60 years.

**(2) WKU KMH**

- At WKU KMH, there was a total of 26,476 patients, including 8,601 patients aged ≥ 60 years (32.4% of all patients), and 61 patients with MCI or dementia (0.2% of all patients) in 2020.

- There was a total of 25,508 patients, including 8,412 patients aged ≥ 60 years (32.9% of all patients), and 41 patients with MCI or dementia (0.2% of all patients) in 2021.

- There was a total of 27,623 patients, including 10,230 patients aged ≥ 60 years (37% of all patients), and 49 patients with MCI or dementia (0.2% of all patients) in 2022.

- Overall, there was an increasing trend in the number of patients aged ≥ 60 years, and while the actual number of patients with cognitive disorders was low, such patients were continuing to visit the hospital during the year.

**(3) DJU DKMH**

- In 2020, there were a total of 16,646 patients, including 4,757 patients aged ≥ 60 years (28.5% of all patients), and 70 patients with MCI or dementia (0.4% of all patients).

- In 2021, there were a total of 16,046 patients, including 4,968 patients aged ≥ 60 years (30.9% of all patients), and 32 patients with MCI or dementia (0.2% of all patients).

- In 2022, there were a total of 14,483 patients, including 4,721 patients aged ≥ 60 years (32.5% of all patients), and 52 patients with MCI or dementia (0.4% of all patients).

- Overall, there was an increasing trend in the number of patients aged ≥ 60 years relative to the total number of patients.

| **Year** | **Category** | **JHIMH** | **WKU KMH** | **DJU DKMH** |
| --- | --- | --- | --- | --- |
| 2020 | Total number of patients | 15,153 | 26,476 | 16,646 |
|  | Number of patients aged ≥ 60 years | 9,598 | 8,601 | 4,757 |
|  | Total number of patient visits for dementia/MCI* | 61 | 1,264 | 432* |
|  | Actual number of patients with dementia/MCI | 19 | 61 | 70 |
| 2021 | Total number of patients | 14,908 | 25,508 | 16,046 |
|  | Number of patients aged ≥ 60 years | 8,264 | 8,412 | 4,968 |
|  | Total number of patient visits for dementia/MCI* | 58 | 517 | 248* |
|  | Actual number of patients with dementia/MCI | 17 | 41 | 32 |
| 2022 | Total number of patients | 22,619 | 27,623 | 14,483 |
|  | Number of patients aged ≥ 60 years | 12,834 | 10,230 | 3,240 |
|  | Total number of patient visits for dementia/MCI* | 92 | 925 | 472* |
|  | Actual number of patients with dementia/MCI | 22 | 49 | 52 |
| * Applicable only to patients with a main diagnosis of dementia/MCI | | | | |

**5. Principal Investigator at Each Center**

| **Name** | **Address** | **PI** | **Telephone** |
| --- | --- | --- | --- |
| WKU JHIMH | 121 Rohaseu-ro, Anyang-myeon, Jangheung, Jeollanam-do, South Korea | Hyung-Won Kang | 061-860-7777 |
| WKU KMH | 895 Muwang-ro, Iksan, Jeollabuk-do, South Korea | Jung-Tae Lim | 063-850-6914 |
| DJU DKMH | 75 Daedeok-daero 176beon-gil, Seo-gu, Daejeon, South Korea | In-Chul Jung | 042-470-9129 |

**6. Study Period**

○ From the date of approval to December 31, 2029.

**7. Study Subjects**

**1) Inclusion criteria**

① Adults aged 55-85 years.

② Individuals diagnosed with a major neurocognitive disorder caused by Alzheimer’s disease, a neurocognitive disorder caused by vascular disease, or a mild neurocognitive disorder based on the criteria described in the Diagnostic and Statistical Manual of Mental Disorders, 5^th^ Edition (DSM-5).

③ Individuals who voluntarily choose to participate or whose legal representatives have consented and signed the consent form.

**2) Exclusion criteria**

① Patients with dementia caused by conditions other than Alzheimer’s disease or vascular dementia (i.e., degenerative brain diseases such as Parkinson's disease, Huntington's disease, frontotemporal disorder, Creutzfeldt-Jakob disease).

② Individuals with systemic conditions that may cause dementia (i.e., hypothyroidism, vitamin B12 or folic acid deficiency, niacin deficiency, hyperkalemia, neurosyphilis, human immunodeficiency virus disease).

③ Individuals with a history of being diagnosed with psychotic disorder or substance-related disorder based on DSM-5 (e.g., schizophrenia, delusional disorder, bipolar disorder, alcohol or substance abuse disorder).

④ Individuals with a history of neurological disorders such as epilepsy, focal brain injury, and head trauma.

⑤ Individuals with gastrointestinal, endocrine, or cardiovascular diseases that cannot be controlled by dietary or drug therapy.

⑥ Individuals in a serious unstable medical condition (as determined by a physician based on laboratory tests, electrocardiogram [ECG], chest X-ray, and vital signs results).

⑦ Others deemed ineligible to participate in the study by the investigator.

**3) Dropout criteria**

① If the subject withdraws consent to participate in the study.

② If the subject is lost to follow-up, such as being unreachable.

③ If the investigator deems the progression of the clinical study inappropriate.

**8. Sample Size and Rationale**

**1) Study population and target enrollment size**

① Recruitment centers: WKU JHIMH, WKU KMH, and DJU DKMH

② Eligibility: Adults aged 55-85 years diagnosed with a major neurocognitive disorder caused by Alzheimer’s disease, a neurocognitive disorder caused by vascular disease, or a mild neurocognitive disorder based on DSM-5 criteria.

③ Target enrollment size: A total of 300 new patients will be competitively recruited between 2024 and 2028 and up to December 2028 at WKU KMH, WKU JHIMH, and DJU DKMH. Each center is expected to recruit approximately 100 candidates, but the number may vary due to the nature of competitive recruitment. At least 800 person-years are expected, assuming one visit between 2024 and 2029 (observation for a minimum of one year to a maximum of six years) and a dropout rate of 30% at 1200 person-years.

2) Sampling method

○ Because this study is a registry study for identifying factors affecting the prognosis of patients with cognitive disorders, the sample size does not need to be calculated in advance since there is no need to test any predetermined statistical hypotheses. However, the sample size will be determined within a realistically possible range considering the funding and circumstances of the study.

○ There is no limitation on the representativeness of the population. Nonetheless, adults aged 55-85 years diagnosed with a major neurocognitive disorder caused by Alzheimer’s disease, a neurocognitive disorder caused by vascular disease, or a mild neurocognitive disorder based on DSM-5 criteria will be selected by WKU KMH, WKU JHIMH, and DJU DKMH, considering realistic applicability.

○ By additionally recruiting 60 new patients over five years, a total of 300 patients will be included. Although registry loss due to mortality and other reasons is expected, the goal is to maintain a retention rate of ≥ 70%.

○ The annual average of the sum of the actual number of patients with dementia/MCI over three years at three centers is 121. Assuming 50% will consent to participate in the study, the target enrollment size is 300 (60 per year over five years). Considering a dropout rate of 30% from a total cumulative 1200 person-years, the target is at least 840 person-years.

○ Patients who do not receive KM treatment may also participate in the study. Patients are not required to receive any KM treatment during the entire study period.

**9. Methods and Procedures**

**1) Methods**

□ Patient enrollment through registry establishment

- A registry study is an observational study designed to collect data through an organized system that continuously collects uniform data and produces meaningful results for assessing the outcomes of populations exposed to a specific disease or factor.

- This study aims to establish a registry for patients with cognitive disorders (e.g., MCI, Alzheimer’s disease, vascular dementia) and collect data on factors that affect cognitive function through annual clinical assessments.

- Subjects who have voluntarily signed a consent form or whose legal representatives have signed on their behalf will be screened based on inclusion and exclusion criteria. Those deemed eligible will be enrolled in the registry through competitive enrollment at WKU KMH, JHIMH, and DJU DKMH.

- To ensure the study’s success, each center will hire a research coordinator responsible for the registry study, and the level of subject recruitment for the registry study at each center will be monitored through a communication system.

□ Registry establishment and problem-coping strategies

- Subjects will visit the study center once a year to undergo a questionnaire survey and physical examination according to the standard operating procedure (SOP), and to receive education for the following visit.

- A major challenge in registry studies is subject follow-up. To minimize dropout rates, materials related to health and cognitive disorders, including information regarding lifestyle, will be distributed at least once a year. Additionally, the study coordinator will contact subjects to monitor their health management status.

□ Biomarker investigation strategy

- After establishing the registry, education on biomarker investigation will be provided to the study coordinator and investigators.

- ECG, chest X-ray, blood sampling, and urinalysis will be performed by investigators affiliated with WKU KMH, WKU JHIMH, and DJU DKMH according to the treatment system. ECG, chest X-ray, and urinalysis will be performed at the center, and blood and urine samples will be analyzed within 24 hours of collection.

**2) Subject Consent and Recruitment**

**1] Informed consent form (ICF) and recruitment announcement**

□ Consent forms for participation in the clinical study and use of personal information

- Prior to obtaining consent to participate in the study, the investigator will use the IRB-approved information sheet and consent form to fully inform subjects who wish to participate. This includes information about the study objectives, use of data, confidentiality, the possibility of data sharing with collaborating centers for analysis, and the right of the subject to withdraw consent at any time. The investigator will also answer questions from the subjects. After signing the ICF, the subject will receive a copy of the ICF.

- Individuals diagnosed with a major neurocognitive disorder based on the inclusion/exclusion criteria will be classified as vulnerable subjects, and consent will be obtained from both the subject and their legal representative. For individuals diagnosed with a mild neurocognitive disorder, consent from only the subject will be obtained. However, additional consent may be obtained if the subject requests re-consent. If the investigator determines that the medical condition of a subject has changed since the initial consent during any follow-up visit, the subject may be reclassified as a vulnerable subject, and additional consent may be obtained from both the subject and their legal representative.

- The capacity of a legal representative to provide consent will be determined by the clinical study physician, and the assessment will be performed through outpatient care. If the investigator determines that the legal representative has a diminished capacity to provide consent, then that subject cannot be enrolled, or consent from a different legal representative will be obtained.

- The legal representative consent form must be completed by the legal representative, and document(s) that can verify the identity of the legal representative (e.g., family relationship certificate) must be obtained and kept.

□ Recruitment method

- The recruitment announcement for this study will be posted on the hospital bulletin board and as a pop-up window on the websites of the recruiting centers. Additionally, the announcement will be posted in public health centers and KM clinics within Iksan. Subjects will also be recruited through local newspapers, mass transportation advertisements, and authorized recruitment sites. The PI (Jung-Tae Lim) will be responsible for the recruitment of all subjects.

- Patients diagnosed with major neurocognitive disorders caused by Alzheimer’s disease, major neurocognitive disorders caused by vascular disease, and mild neurocognitive disorders will be informed about the study. Patients who voluntarily agree to participate will receive a detailed explanation and a written information sheet. Patients who ultimately decide to participate and submit an ICF will be screened for eligibility based on the inclusion/exclusion criteria. Eligible patients will be enrolled in the study.

□ Voluntary participation and withdrawal of consent

- Subjects will be informed that participation in the study is voluntary and that there will be no negative consequences for choosing not to participate. Subjects may withdraw their consent at any time during the study by immediately notifying the PI or the investigator in charge. Even if consent is withdrawn, data collected up to that point may still be accessed, processed, and used for purposes related to the clinical study.

**2] Recruitment and enrollment methods**

- Subjects will be assigned a screening number in the order in which the ICF was obtained, recorded as follows:

- Study center code: WKU JHIMH (WJH), WKU KMH (WKH), and DJU DKMH (DJH)

- Study center code–Year–First letter of “Screening”–Order of enrollment

(e.g., WKH-2023-S-015: 15^th^ patient screened in 2023 at WKH)

- Subject identification code will be recorded as follows:

- Study center code: WJH, WKH, and DJH

- Study center code–Year–First letter of “Enrollment”–Order of enrollment

(e.g., WKH-2023-E-015: 15^th^ patient enrolled in 2023 at WKH)

- The name of each subject will appear as initials.

| **Subject identification code** | **Subject initials** |
| --- | --- |
| \|  \|  \|  \| \| --- \| --- \| --- \|   **– 202_ - S -**   \|  \|  \|  \| \| --- \| --- \| --- \| | \|  \|  \|  \| \| --- \| --- \| --- \| |
| \|  \|  \|  \| \| --- \| --- \| --- \|   **– 202_ - E -**   \|  \|  \|  \| \| --- \| --- \| --- \| |  |
| □ NA (Screening Fail) |  |

**3) Procedures**

**1] Visits and intervals**

- There will be one visit per year, from the time of enrollment to December 2029. Patients enrolled in the second year (2024) will be observed for six years, while those enrolled in the sixth year will be observed for two years.

| Screening | Consent | - Subject and legal representative consent forms |
| --- | --- | --- |
|  | Screening tests | - Demographic survey, medical and medication history taking - Vital signs, height, and body weight - Laboratory tests - Questionnaire survey for patient inclusion/exclusion |
| Visit  1 | Baseline tests | - Basic test (PIT-C) - Long-term care service utilization status - Guardian questionnaire survey - Patient questionnaire survey - Instrument tests - Confirmation of disease and treatment history; concomitant drugs - Education on the visit schedule |
| Visit  2~6  (common) | Follow-up tests | - Laboratory tests - Vital signs, height, and body weight - Long-term care service utilization status - Guardian questionnaire survey - Patient questionnaire survey - Instrument tests - Confirmation of disease and treatment history; concomitant drugs - Education on the visit schedule |

**2] Survey content**

(1) Screening tests

① Demographic characteristics (smoking history, drinking history, family medical history, education level, literacy, occupation, marital status, driving status, telephone use status, guardian information, health insurance status, care cost, the burden of the hospital bill, sex and age of guardian, address, telephone number, occupation, past and present medical history, care burden, relationship to the patient, cohabitation status, time spent for care, average monthly income, monthly expenses of the patient, cognitive therapy status, insurance related to cognitive disorders, etc.) and medical and medication history (medical history for the past three years, present medical history, family history or personal history of dementia or stroke, lifestyle, and medication history) are surveyed and vital signs, height, and body weight are measured.

② Laboratory tests: Clinicopathologic and additional tests may be performed as needed (* performed only during screening).

□ WKU JHIMH and WKU KMH (performed by in-house laboratory)

- Hematology tests: WBC, RBC, hemoglobin, hematocrit, platelet, MCV, MCH, MCHC, and fibrinogen*

- Blood chemistry tests: Glucose, BUN, creatinine, AST, ALT, ALP, γ-GTP, total bilirubin, albumin, total protein, total cholesterol, triglyceride, HDL/LDL cholesterol, TSH, free T4, CRP, vitamin B12*, folate*, homocysteine*, TPHA*, and VDRL* (If TPHA and VDRL tests cannot be performed, syphilis test is permitted to rule out syphilis).

- Urinalysis: Specific gravity, nitrite, pH, protein, glucose, ketone, urobilinogen, bilirubin, WBC, and RBC.

- ECG and chest X-ray

□ DJU DKMH (performed by in-house laboratory)

- In the list of items above, VDRL and TPHA are replaced with syphilis tests.

③ Patient questionnaire survey: Cognitive function (K-MMSE2, MoCA-K, and GDS) and depression (S-GDps)

(2) Baseline tests (Visit 1)

① Basic test (PIT-C)

② Guardian questionnaire survey

A. Patient-related assessment: Patient’s activities of daily living (K-BADL, S-IADL) and aberrant behavior (NPI-Q)

B. Guardian-related assessment: Care burden of primary caregiver

③ Patient questionnaire survey: QoL (GQoL-D, EQ-5D-5L, EQ-VAS), blood debilitation scale, and Core Seven Emotions Inventory-Short Form (CSEI-s).

④ Long-term care service utilization status: Home health care, home bathing, home nursing, day or overnight care, short-term care, and facility services.

⑤ Changes in medical and treatment history and concomitant drugs.

⑥ Instrument tests

A. Mandatory: HRV

B. Optional depending on the patient’s condition: QEEG and fNIRS.

⑦ Education on the following visit.

(3) Follow-up tests (Visit 2 – Visit 6)

- Follow-up tests will be performed once a year after the baseline tests.

- Changes in disease and treatment history and concomitant drugs; measurement of vital signs, height, and body weight; laboratory tests, guardian questionnaire survey, patient questionnaire survey, long-term care service utilization status, instrument tests, and education on the following visit.

Study flow chart

| **Visit**^1)^ | | **Screening** | **1** | **2** | **3** | **4** | **5** | **6** |
| --- | --- | --- | --- | --- | --- | --- | --- | --- |
| **Year** | |  | **0** | **1**  **(±30일)** | **2**  **(±30일)** | **3**  **(±30일)** | **4**  **(±30일)** | **5**  **(±30일)** |
| Obtain subject and legal representative consent forms | | ● |  |  |  |  |  |  |
| Confirm inclusion/exclusion criteria | | ● |  |  |  |  |  |  |
| Assign screening number | | ● |  |  |  |  |  |  |
| Demographic survey^2)^ | | ● |  |  |  |  |  |  |
| Medical and medication history taking^3)^ | | ● |  |  |  |  |  |  |
| Vital signs, height, and body weight^4)^ | | ● |  | ● | ● | ● | ● | ● |
| Laboratory tests^5)^ | | ● |  | ● | ● | ● | ● | ● |
| Long-term care service utilization status | |  | ● | ● | ● | ● | ● | ● |
| PIT-C | |  | ● |  |  |  |  |  |
| Guardian | Care burden of the primary caregiver |  | ● | ● | ● | ● | ● | ● |
| Patient condition (checked by the guardian) | K-BADL |  | ● | ● | ● | ● | ● | ● |
|  | S-IADL |  | ● | ● | ● | ● | ● | ● |
|  | NPI-Q |  | ● | ● | ● | ● | ● | ● |
| Patient condition | K-MMSE2 | ● |  | ● | ● | ● | ● | ● |
|  | MoCA-K | ● |  | ● | ● | ● | ● | ● |
|  | GDS | ● |  | ● | ● | ● | ● | ● |
|  | S-GDpS | ● |  | ● | ● | ● | ● | ● |
|  | CSEI-s |  | ● | ● | ● | ● | ● | ● |
|  | Blood debilitation scale |  | ● | ● | ● | ● | ● | ● |
|  | EQ-5D-5L |  | ● | ● | ● | ● | ● | ● |
|  | EQ-VAS |  | ● | ● | ● | ● | ● | ● |
|  | GQOL-D |  | ● | ● | ● | ● | ● | ● |
| Instrument tests  (mandatory) | HRV |  | ● | ● | ● | ● | ● | ● |
| (Optional depending on the patient’s condition) | QEEG |  | ● | ● | ● | ● | ● | ● |
|  | fNIRS |  | ● | ● | ● | ● | ● | ● |
| Changes in disease and treatment history and concomitant drugs | |  | ● | ● | ● | ● | ● | ● |
| Education on the visit schedule | |  | ● | ● | ● | ● | ● | ● |

1) Visit 1 should occur within 10 days of the screening visit. If test results are available on the same day, the Visit 1 test may be performed. Visits 2–6 should be scheduled within ±30 days of the date of Visit 1 in each subsequent year.

2) Demographic survey: Demographic information (subject initials, sex, date of birth, telephone number, address, education level, and occupation) is collected.

3) Medical and medication history taking: Medical history for the past three years, present medical history, and medication history are recorded.

4) Vital signs, height, and body weight: Body temperature, blood pressure (systolic/diastolic), and pulse rate are measured.

5) Clinicopathologic tests including the following and additional tests may be performed as needed (* performed only during screening).

□ WKU JHIMH and WKU KMH (performed by in-house laboratory)

- Hematology tests: WBC, RBC, hemoglobin, hematocrit, platelet, MCV, MCH, MCHC, and fibrinogen*

- Blood chemistry tests: Glucose, BUN, creatinine, AST, ALT, ALP, γ-GTP, total bilirubin, albumin, total protein, total cholesterol, triglyceride, HDL/LDL cholesterol, TSH, free T4, CRP, vitamin B12*, folate*, homocysteine*, TPHA*, and VDRL* (If TPHA and VDRL tests cannot be performed, syphilis test is permitted to rule out syphilis).

- Urinalysis: Specific gravity, nitrite, pH, protein, glucose, ketone, urobilinogen, bilirubin, WBC, and RBC

- ECG and chest X-ray

□ DJU DKMH (performed by in-house laboratory)

- In the list of items above, VDRL and TPHA are replaced with syphilis tests.

○ Details of questionnaire survey items

(1) Activities of daily living

① Seoul-Instrumental Activities of Daily Living (S-IADL)

- The S-IADL was developed and standardized with items suitable for the cultural characteristics of Korea. It consists of 15 items rated on a 0-3 point scale, with a total score ranging from 0 to 45 points. Higher scores indicate greater disability in daily life. The scale differentiates IADL into “current ability” and “potential ability.” “Current ability” assesses whether the patient can independently perform IADL at present, while “potential ability” assesses whether the patient has the potential to perform IADL even if unable to do so independently now. This scale has excellent reliability and validity and can differentiate healthy individuals from patients with dementia based on a cutoff score of 7.5 points (≥ 8 points indicating dementia).

② Korean version of Barthel Activities of Daily Living (K-BADL)

- The K-BADL assesses the ability to perform basic activities of daily living. The scale is based on a total score of 20 points, with 11–15 points indicating moderate disability and ≤ 10 points indicating severe disability.

(2) Aberrant behavior

① Brief Questionnaire form of the Neuropsychiatric Inventory (NPI-Q)

- The NPI-Q was developed for easy use in clinical practice. All 12 items have a high correlation with NPI (r=0.71-0.93), but NPI-Q is quicker to complete, taking less than 5 minutes compared to NPI, which takes more than 15 minutes. NPI-Q differs from NPI in several ways. Firstly, it does not use an interview format but a questionnaire survey format where the guardian reads the items and marks the responses. Secondly, it includes screening questions for 12 aberrant behaviors but no specific questions. Thirdly, it assesses the severity of aberrant behaviors and caregiver distress but not the frequency. This study will use the translated Korean version of NPI-Q.

(3) Quality of Life (QoL)

① Health-related QoL scale (EuroQoL-5 Dimension 5 Level; EQ-5D-5L)

- The EQ-5D was developed and successively improved by the EuroQol Group, which was established in 1987. The Korean EQ-5D (KEQ-5D) is a multidimensional preference-based health-related QoL (HRQoL) measure that measures HRQoL based on utility. Recently, the EuroQol Group introduced a new QoL scale with each domain in EQ-5D-3L expanded from three to five levels to overcome the shortcomings of EQ-5D-3L. While EQ-5D-3L could express 243 health states, EQ-5D-5L can express 3,125 (=55) health states. EQ-5D-5L is expected to reduce the ceiling effect of EQ-5D-3L and improve technical richness, enhancing reliability and sensitivity. A study comparing EQ-5D-3L and EQ-5D-5L in cancer patients from a Korean hospital found that EQ-5D-5L had a lower ceiling effect, higher validity, and similar test-retest reliability compared to EQ-5D-3L. The EuroQol Group currently provides the official Korean version of EQ-5D-5L.

② General health state score (EuroQoL Visual analogue scale; EQ-VAS)

- The EQ-VAS is a 20 cm vertical visual analogue scale used as a rating scale. A score between 0 (worst imaginable health) and 100 (best imaginable health) is assigned to a given health state. The score clearly indicates the order of health outcomes and provides information about the strength of preference. However, measurement bias can occur when using a rating scale. Rating scales are typically recommended as auxiliary tools to other tools (National Evidence-based Healthcare Collaborating Agency, 2013). EQ-VAS is included as an auxiliary tool in EQ-5D-5L and EQ-5D-3L standard tests.

③ Geriatric Quality of Life-Dementia (GQOL-D)

- The GQOL-D consists of 15 items (13 items on physical health, psychological health, social relations, and environment; one item on overall health; and one item on overall life satisfaction), with each item rated on a 3-point Likert scale. The total score, ranging from 15-120 points, is the sum of the responses to each item. The total score is converted to a normative score (T-score) that accounts for sex and age. Higher T-scores indicate higher subjective QoL or life satisfaction. A T-score ≤ 35 points indicates low QoL.

(4) Mini Mental Status Examination (MMSE)

- The MMSE is one of the most widely used screening tests, a simple test developed by Folstein et al. (1975), with a total score of 30 points and requires 5–15 minutes to complete. Its low learning effect allows for examining changes over time based on repeated measures during disease progression (Folstein et al, 1975). Moreover, its reliability and validity have been proven for screening patients with moderate and severe dementia (Kaszniak et al., 1986) and is widely used in Korea.

- In Korea, the K-MMSE adapted by Kang et al. (1997) and MMSE-K adapted by Kwon et al. (1989) are commonly used. This study will use the K-MMSE, which has a total score of 30 points, including orientation for time (5 points), orientation for place (5 points), memory registration (3 points), attention and calculation (5 points), memory recall (3 points), language ability (8 points), and visuospatial construction ability (1 point). While the MMSE is easy to apply with simple training, its results may be influenced by differences in education level, age, culture, and language. It lacks items to assess frontal lobe functions, making it difficult to differentiate frontotemporal and vascular dementia accurately, and also makes differentiation between mild and severe memory impairment challenging due to the narrow range of difficulty levels.

- A total score of 23 points is generally considered the cutoff point for cognitive impairment. Epidemiological studies classify total MMSE scores of 24–30 points as no cognitive impairment, 18-23 points as MCI, and 0-17 points as clear cognitive impairment.

(5) Montreal Cognitive Assessment-Korean (MoCA-K)

- The MoCA-K is a tool developed to screen for MCI among individuals with normal findings in the MMSE. It assesses short-term memory, visuospatial skills, executive function, concentration and working memory, language, and orientation, and takes approximately 10 minutes to complete. A total score of ≥ 23 points is considered normal. The MoCA-K is not recommended for individuals who cannot read or write or have difficulty in these areas.

(6) Short form Geriatric Depression Scale (S-GDpS)

- The S-GDpS is a short form (15 items) of the Geriatric Depression Scale (GDpS) developed into a Korean version by Cho et al. for assessing geriatric depressive symptoms. A score of 5 points indicates suspected depression.

(7) Global Deterioration Scale (GDS)

- The GDS is a dementia-related deterioration scale commonly used in Europe, which has been translated into Korean and standardized. GDS 1 represents clinically normal with no cognitive impairment, GDS 2 represents subjective memory impairment, and GDS 3 represents MCI. Some patients with mild dementia may also be included in GDS 3. GDS 4 and above clearly indicate dementia; GDS 4 for mild dementia, GDS 5 for moderate dementia, and GDS 6 and 7 for severe dementia.

(8) Blood Debilitation Scale

- The Blood Debilitation Scale is a scale for assessing pathologic aging in KM, included as an assessment tool in KM doctor dementia diagnosis report writing guidelines (long-term care grade 5). It consists of aging-related sensory and physical symptoms and has a total of nine items. Each item is rated as never (0 point), sometimes (1 point), and often (2 points), with lower scores indicating a more favorable blood debilitation level.

(9) Core Seven-Emotions Inventory Short Form (CSEI-s)

- The CSEI-s consists of 28 items: four items each for joy, anger, thought, depression, sorrow, fear, and fright. Each item is rated on a 5-point Likert scale (1=never, 2=rarely, 3=somewhat, 4=often, and 5=almost always).

- This scale uses T-scores (mean: 50 points, standard deviation: 10 points). Higher scores for anger, thought, depression, sorrow, fear, and fright, but not joy, indicate the high-risk group. The specific cutoff points are T-scores of 55-60 points for the caution group, 61-65 points for the risk group, and ≥ 66 points for the high-risk group. Lower scores for joy represent the risk group, with 40-45 points for the caution group, 35-39 points for the risk group, and ≤ 34 points for the high-risk group (Note: Emotion cards may be used as an alternative for patients with severe dementia. Elderly individuals with dementia do not have significant difficulty in expressing their internal state if their MMSE-K score is ≥ 10 points, even with cognitive impairment).

(10) Pattern Identifications Tool for Cognitive Disorders (PIT-C)

- A clinical study assessing the reliability and validity of the PIT-C was conducted as part of the development of KM standard practice guidelines for dementia, sponsored by the Korea Health Industry Development Institute. The study reported not only high reliability but also correlations with other cognitive disorder assessment tools. This tool can determine the top pattern identifications and quantify the tendencies of other pattern identifications. Consequently, it can be used for selecting the main prescription for herbal medicine and acupuncture therapy, adjusting the number of herbs, selecting acupuncture points for concomitant acupuncture therapy, and assessing treatment progress.

**3] Adverse reactions**

- As this study is a registry study with no intervention, the expected risks and adverse events are determined to be low. All medical history newly collected during follow-up visits after the first visit will be recorded as medical history, not adverse reactions. Only the death of a subject will be collected and recorded as an adverse reaction. Deaths caused by participation in the study (deaths associated with the study) are not anticipated, but since the study population consists of the elderly, deaths may occur during the study. Therefore, the cumulative number of deaths for all sites will be pooled and reported together during the continuous review by each center.

**10. Data Collection and Management**

○ Data collection method and database construction

- Data for this study will be collected from paper case records submitted for the study, compiled, and inputted into an electronic case record file (e-CRF) developed and managed by the National Development Institute of Korean Medicine. Subsequently, a database will be constructed through the data-cleaning process, and analyses will be performed.

○ Collection of medical records, questionnaire responses, and electronic data from medical institutions

- The procedures for collecting medical records, questionnaire responses, and electronic data from participating medical institutions are as follows:

1) Obtain review and approval from the IRB of each participating medical institution.

2) Define research and data collection methods in consultation with participating investigators and personnel responsible for investigating medical records, questionnaire responses, and electronic data at each medical institution.

3) Assign patient enrollment numbers on an institutional and individual patient basis to all subjects selected according to the inclusion criteria.

4) Store originals of supporting documents or worksheets containing results from an investigation of medical records, questionnaire responses, and electronic data based on patient enrollment numbers at each medical institution. The PI will remotely access the e-CRF to input the case records.

○ Data monitoring and quality control

- Monitoring will be conducted by a contract research organization (CRO; ariBnC).
- General monitoring procedures will comply with relevant regulations and the monitoring plan. Specific monitoring plans will follow the attached monitoring plan (Appendix 01. Monitoring Plan).

**11. Data Analysis**

This registry study does not have a predefined primary outcome and will utilize the following analysis methods:

○ Nominal variables will be presented as frequency and ratio, while continuous variables will be presented as mean and standard deviation.

○ Changes in continuous variables in all patients or specific subgroups will be tested using either the paired t-test or the Wilcoxon signed rank test, depending on the normality test results. Changes in nominal variables will be tested using McNemar’s test. Correlations of continuous variables will be analyzed using Pearson’s (or Spearman’s) correlation analysis.

○ Multinomial regression analysis will be used to test the effects of covariates on a particular continuous dependent variable, while multinomial logistic regression analysis will be used to test the effects of covariates on the occurrence of binominal indicators. Survival analysis will be performed to test the effects of covariates on the occurrence of a particular binomial variable over time.

○ Differences in continuous variables between particular subgroups will be analyzed using an independent t-test (or Wilcoxon rank sum test, depending on the normality test results). Differences in nominal variables will be analyzed using the chi-square test (or Fisher’s exact test, depending on the normality test results).

**12. Subject Compensation**

○ Each subject will receive 100,000 won for each visit as compensation for participating in the study (for transportation costs). Patients enrolled in the second year who complete all six visits will receive up to 600,000 won (100,000 won x 6), while patients enrolled in the sixth year who complete two visits will receive up to 200,000 won (100,000 won x 2). The payment will be made during each visit, not as a lump sum payment, and will not be contingent on the completion of the study.

**13. Protection of Privacy and Handling of Personal Information**

(1) Familiarization with the protocol

○ This study has been prepared in consideration of the rights and welfare of the study subjects in accordance with the Declaration of Helsinki. The PI and other investigators are responsible for accurately analyzing and being familiar with the protocol; prioritizing the health and human rights of the study subjects; and actively participating in efforts to resolve any problems that may occur during the clinical study.

(2) Protocol compliance and amendment

○ The clinical study will be conducted in accordance with the approved protocol. Any amendment to the protocol must be discussed with the sponsor and the amended protocol will be prepared by the sponsor. The investigators may not apply the modifications until such modifications have been reviewed and approved by the IRB, except when necessary to prevent immediate harm to the study subjects. Any serious protocol deviations will be documented in the case report.

○ If protocol amendments or modifications are applied prior to IRB approval to prevent immediate harm to the study subjects, information regarding such amendments or modifications must be submitted to the IRB (for post-hoc review), the sponsor, and relevant ministries (if required by relevant regulations) as soon as possible. If the protocol modification is minor, the investigator notifying the IRB may suffice. However, in the event of fundamental change to the study design or potential harm to the study subjects, the investigator must:

1) Revise the ICF and submit it to the IRB for review and approval

2) Re-consent using the revised ICF must be obtained from the subjects who have already been enrolled

3) Use the revised ICF to obtain consent from newly recruited subjects.

(3) Subject consent

○ Prior to the start of the clinical study, subjects must be fully informed about the content and safety of the clinical study, and an ICF indicating voluntary consent to participate in the study must be obtained. The subject consent process shall adhere to the ethical principles prescribed in the Declaration of Helsinki.

○ The investigator must provide a copy of the ICF and a copy of the information sheet containing written information about the clinical study in non-technical, easy-to-understand terms to each subject or their legal representative. The investigator will give sufficient time to the subject or their legal representative to ask questions about the specific details of the clinical study. After this, the subject or their legal representative will personally sign and date the ICF. When necessary, another person who also discussed the study during the consent process may also sign. Each subject or their legal representative must receive a copy of the signed ICF and a copy of the information sheet before participating in the study.

○ If the subject or their legal representative is illiterate, an impartial witness must take part in the entire consent process. If the subject or their legal representative can verbally consent and sign, the impartial witness will sign and date the form to confirm that the information contained in the ICF was accurate, the subject or their legal representative understood the information, and the consent was given by free will.

○ The ICF and other information provided to the subject or their legal representative must be updated whenever new information becomes available that is material to the subject’s consent. The IRB’s opinion must be considered before making any updates. The investigator must fully inform the subject or their legal representative about any new information related to all valid aspects of the clinical study, and document the subject’s decision to participate in the study. Such interactions must be documented and retained.

○ In the event of an inspection by the study center, research funding organization, and/or public IRB designated by the Ministry of Health and Welfare, I consent to direct access to my personal information held in confidence.

○ In addition, since this study is a government-funded project of the National Development Institute of Korean Medicine, data from this study may be anonymized and linked to secondary data sources, such as national health insurance claims data, for future use. Accordingly, separate consent will be obtained for granting access to third parties and for use in secondary research. Information provided for secondary research will be stored, managed, and provided from the completion of the primary study until the destruction of information is requested or up to 10 years later. Personal information that has reached the end of its retention period will be destroyed in accordance with Article 16 of the Enforcement Decree of the Personal Information Protection Act. The personal and sensitive information collected in this study includes the following:

**(Personal information) Name, date of birth, sex, address, and mobile telephone number.**

**(Sensitive information) Health-related information, including Korean Classification of Diseases (KCD) code, height, body weight, smoking status, drinking status, education level, occupation, past history, medication history, surgery history, family history, medical history, vital signs, pattern identification, test results, and questionnaire responses.**

Accordingly, the investigator shall fully inform the subject or their legal representative about the right to personal information protection guaranteed under the Personal Information Protection Act. The subject or their legal representative shall, of their free will, consent to the disclosure of documents or materials to the sponsor and third parties in related organizations, as requested in this study among personal information. The sponsor shall provide the consent forms for personal information collection, use, and provision to the PI in accordance with the Personal Information Protection Act, and the PI shall distribute the consent forms to the subjects and their legal representative to obtain their consent. Therefore, one set each of an information sheet and a consent form for disclosure to third parties and use in secondary research (hereinafter secondary research use consent form) will be provided separately.

(4) Accurate selection of subjects

○ Prior to the start of this clinical study, the eligibility of the subjects will be thoroughly assessed through sufficient investigation.

(5) Clinical study audit

○ The PI shall regularly check the progress, circumstances, and results of the clinical study, and the IRB may conduct an audit on the progress of the clinical study when necessary.

(6) Monitoring of study centers

○ Monitoring will be conducted to ensure that the rights and welfare of the subjects are protected; reported data related to the clinical study are accurate, complete, and verifiable against supporting documents; and the clinical study is being conducted according to the approved protocol, Korean Good Clinical Practice guidelines, and enforcement rules. Monitoring of the clinical study may be conducted by a designated monitor through regular visits to the study centers or via telephone. During each visit, the monitor will check the original records of the subjects, procedure management records, and data storage. In addition, the monitor will carefully review the progress of the clinical study and discuss any problems with the investigators. Monitoring in this study will be conducted by an investigator from WKU KMH and a designated monitor.

(7) Confidentiality

○ All personally identifiable records will be kept confidential, and the identity of the subjects will remain confidential even if the clinical study results are published. The sponsor, monitor, and auditor associated with this study may access the records of the subjects for the purposes of monitoring, auditing, and managing the progress of this study. Once the contract for this clinical study is signed, the investigator shall be aware that the sponsor, monitor, or auditor of the CRO may review or copy relevant documents to verify the chart and CRF records of the subjects. The subject identification code (usually a sequential number assigned or initials), not the name, shall be used in all documents related to the clinical study, including the CRF.

(8) Data results and reporting

○ The investigator shall prepare and maintain case details in an appropriate and accurate manner in the CRF designed to record all necessary data, including all observational results for each subject participating in the study.

○ Data derived from supporting documents that are entered in the CRF must be consistent with the supporting documents, and all inconsistencies must be explained.

○ The CRF must be written legibly with a pen. The subjects shall be identified by their English name initials, date of birth, and subject identification number. All required information must be entered in the applicable fields in the CRF. If the information is not available or not applicable, it should be stated as such, and no field should be left blank. Personally identifiable records of the subjects should be kept confidential to ensure the privacy of the subjects is protected in accordance with the relevant regulations.

○ When revising data, incorrect entries should be crossed out, and accurate information should be written next to it. The initials of the person who made the revision and the date should be written, and the reason for the revision should be written when necessary.

○ Completed CRFs shall be reviewed in a timely manner, signed, and dated by the investigator or a physician who is qualified as an investigator at the study center. The investigator must keep a copy of the CRF that includes all changes and revisions.

(9) IRB

○ Prior to the start of the study, the investigator must obtain written approval from the IRB for the protocol, ICF, data and procedures related to subject recruitment, and written information sheet to be provided to the subjects. The investigator must also provide the IRB with the clinical study report, the latest information, and other information (e.g., safety updates, modifications, notifications) in accordance with the relevant regulations and in-hospital procedures.

**14. Expected Risks and Benefits to the Subjects**

○ The subjects will participate in questionnaire surveys during the follow-up process, which will require approximately 120 minutes to complete.

○ As a registry study, the expected risks in this study are very low. Nonetheless, common reactions such as pain, discomfort, bruising, and fatigue may occur during the blood sampling process, with rare adverse reactions such as numbness, hematoma, infection, and nerve damage.

○ There is no direct financial benefit to the subject for participating in this study. However, the subject may benefit indirectly from receiving quality healthcare service for the target disease through the establishment of the KM treatment registry for that particular disease.

○ The subjects may undergo various physical examinations during the program.

**15. Safety Protection Measures and Indemnification**

○ In the event of an unscheduled visit, the reason for the visit, medical history, changes in medication, and vital signs shall be recorded in the CRF.

○ To minimize the possibility of coercion or undue influence, subjects must be notified about the voluntary nature of their participation, their right to refuse or discontinue participation without negative consequences, and their right to withdraw consent.

○ Clinical study liability insurance will be maintained.

○ Matters related to the protection of vulnerable subjects:

- Reason for including vulnerable subjects: This study involves a patient registry that manages the diagnosis, treatment, and prognosis of patients with cognitive disorders (MCI, Alzheimer’s disease, and vascular dementia), which may include adults with limited capacity to consent.

- Procedures for minimizing risks: Subjects will be assessed for their capacity to consent and informed about their right to withdraw at any time without negative consequences.

- Plan for assessing capacity to consent: Assessment based on 1) whether the subject understands information related to the study; 2) whether the subject can logically process information related to the study; and 3) whether the subject is capable of clearly expressing their own choice regarding participation in the study.

- Plan for surrogate consent: If obtaining consent from the subject is difficult, written authorization from the subject and consent from the legal representative will be obtained.

- Plan for consent from the subject: If the subject has sufficient capacity to consent, consent will be obtained directly from the subject.

**16. Data Storage**

○ All data collected and acquired during this study shall only be used for the purposes of conducting the study and writing the study report. Subject data will be stored securely to prevent loss and theft. The investigator in charge of data storage will be Jung-Tae Lim. Upon completion of the study, data will be stored securely in the document storage cabinet in the laboratory of the WKU KMH clinical research center and will be destroyed after three years. However, the retention period may be extended if deemed necessary by the sponsor.

○ Procedures for destroying data and records collected during this study: Information recorded and stored electronically will be destroyed irreversibly, while paper documents will be shredded and pulped.

**17. References**
