# Supplementary material for: Korean medicine registry for cognitive disorder: A protocol for prospective observational multi-center study
Source: PLoS One. 2025 May 15;20(5):e0323170. doi: 10.1371/journal.pone.0323170 (PMC12080776; doi:10.1371/journal.pone.0323170)
Supplement: S3 File — (DOCX) [file pone.0323170.s003.docx]

연구계획서

**인지장애 한의 레지스트리**

**Korean medicine registry for cognitive disorder**

| Protocol No. | WKH-CIR-2023 |
| --- | --- |
| Version | 1.2  (Date of Written : 2024. 01. 22.) |
| 임상연구 실시기관 | 원광대학교 한방병원 |
| 공동연구기관 | 원광대학교 장흥통합의료병원  (책임자: 강형원; 전체 연구과제 PI)  대전대학교 대전한방병원  (책임자: 정인철) |
| 의뢰기관 | 연구자 주도 임상연구로 해당없음 |
| 실시기관 연구책임자 | 임정태 |

| ***CONFIDENTIAL*** |
| --- |
| 본 연구 계획서와 관련된 모든 정보는 기밀 사항이며,  사전 서면 동의 없이는 정보를 유출할 수 없습니다. |

**▣ 임상연구 계획서 제·개정 이력**

| **No** | **Version No.** | **Version Date** | **주요 내용** |
| --- | --- | --- | --- |
| 1 | 1.0 | 2023. 10. 26. | 임상연구 계획서 최초 제정 |
| 2 | 1.1 | 2023. 10. 30. | 원광대학교 한방병원 IRB 제출전 타 기관 수정사항 반영하여 Ver 1.1으로 수정 |
| 3 | 1.2 | 2024. 01. 22. | 원광대학교 한방병원 IRB, 대전대학교 대전한방병원 IRB 심의의견 반영하여 Ver 1.2으로 수정 |

**Protocol Agreement**

인지장애 한의 레지스트리

Korean medicine registry for cognitive disorder

임상시험계획서번호: WKH-CIR-2023

계획서 버전: 1.2

최종 작성일: 2024.01.22.

- 본인은 임상시험계획서를 숙지하였으며, 모든 규정과 지침서에 따라 본 시험을 수행할 것을 동의합니다.
- 본인은 이 임상시험계획서와 관련하여 개발하거나 얻은 모든 정보의 보안을 유지할 것을 동의합니다.

|  |  |  |
| --- | --- | --- |
| **Principal Investigator**  원광대학교 한방병원 임정태 |  | **Date** |

**Protocol Agreement**

인지장애 한의 레지스트리

Korean medicine registry for cognitive disorder

임상시험계획서번호: WKH-CIR-2023

계획서 버전: 1.2

최종 작성일: 2024.01.22.

- 본인은 임상시험계획서를 숙지하였으며, 모든 규정과 지침서에 따라 본 시험을 수행할 것을 동의합니다.
- 본인은 이 임상시험계획서와 관련하여 개발하거나 얻은 모든 정보의 보안을 유지할 것을 동의합니다.

|  |  |  |
| --- | --- | --- |
| **Principal Investigator**  원광대학교 장흥통합의료병원 강형원  (전체 연구과제 PI) |  | **Date** |

**Protocol Agreement**

인지장애 한의 레지스트리

Korean medicine registry for cognitive disorder

임상시험계획서번호: WKH-CIR-2023

계획서 버전: 1.2

최종 작성일: 2024.01.22.

- 본인은 임상시험계획서를 숙지하였으며, 모든 규정과 지침서에 따라 본 시험을 수행할 것을 동의합니다.
- 본인은 이 임상시험계획서와 관련하여 개발하거나 얻은 모든 정보의 보안을 유지할 것을 동의합니다.

|  |  |  |
| --- | --- | --- |
| **Principal Investigator**  대전대학교 대전한방병원 정인철 |  | **Date** |

**목차**

**1. 임상 연구의 명칭** **6**

**2. 연구 배경** **6**

**3. 연구 목적 10**

**4. 연구 기관 및 주소** **11**

**5. 기관별 연구책임자** **12**

**6. 연구 기간** **12**

**7. 연구 대상자** **13**

**8. 연구 대상자 수와 산출 근거** **13**

**9. 연구 방법 및 절차** **14**

**10. 자료 수집 및 관리** **25**

**11. 자료 분석 방법** **25**

**12. 연구 대상자 보상** **26**

**13. 연구 대상자의 사생활 보호, 개인정보 보호 및 처리** **26**

**14. 연구 대상자에게 기대되는 위험과 이익** **29**

**15. 연구 대상자의 안전보호대책과 보상방법 등** **30**

**16. 자료의 보관** **31**

**17. 참고문헌** **32**

**1. 임상 연구의 명칭**

국문명 : 인지장애 한의 레지스트리

영문명 : Korean medicine registry for cognitive disorder

**2. 연구 배경**

□ 2025년도 초고령화 사회 진입, 2060년 인구의 43.9%가 노인이 될 것으로 예상

○ 통계청 발표에 따르면 우리나라는 2018년 ‘고령사회’에 진입한 지 7년 만인 2025년 ’초고령사회‘에 진입. 2060년에는 인구의 43.9%가 노인이 될 것으로 예상

○ 생산연령 인구(15～64세)의 감소로, 100명당 65세 이상 인구를 뜻하는 노년 부양비가 ’20년 21.7명이지만 ‘36년 51.0명, ’60년에는 91.4명에 이를 것으로 전망
- 75세 이상 노인들 적자를 메우기 위해 기초연금을 포함 정부 지원 규모가 2010년 12조 8340억 원에서 2016년 28조 9600억 원으로 증가

[65세 고령인구 증가추이]


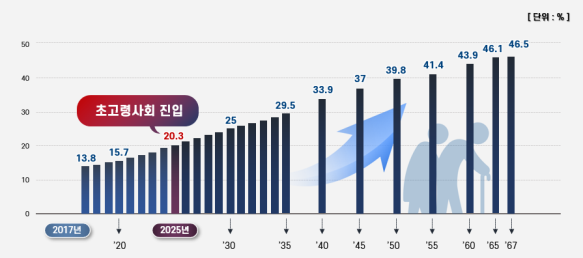


□ 2018년 노인 진료비가 전체 진료비의 40%를 넘어, 2025년 65세 이상 노인 진료비가 60조원에 육박할 것으로 추산

□ 초고령화 시대, 건강하게 늙고 삶의 질을 향상시키기 위한 노화 연구 수행 필요

○ 노화 관련 질환 치료제의 효능이 낮고, 미리 예방하고 관리하여 건강한 상태로 나이가 드는 ‘건강노화’*에 대한 요구 증대
*WHO는 ‘건강노화 2020-2030 계획’을 공표, ‘건강노화’란 낮은 질병 발생률과 높은 인지 및 신체적 기능 그리고 활발한 사회활동 참여가 가능한 노화 상태를 말하는 것으로, 정상노화과정에 노화 관련 질환이 동반되는 ‘일반 노화’와 대조되는 개념

○ 세계적으로 인구 고령화가 진행되면서 노화 연구는 새로운 성장 산업으로 대두되고 있는 상황
- 세계 항노화 및 서비스 시장이 2017년 625억 달러로 연평균 6.5% 성장해 2022년 886억 달러(109조) 규모로 커질 것으로 예상

□ 2019년 국내 65세 이상 치매인구 78만 8,000명

○ 복지부 중앙치매센터에 따르면 2019년 기준 국내의 65세 이상의 치매인구는 78만 8,000명으로서 점점 증가하고 있음, 이에 따라 치매 관리비용은 10년마다 2배씩 증가하여, 2050년에는 치매로 인한 사회적 비용이 약 78조 원에 이를 것으로 전망하고 있음. 이에 정부는 치매 예방 및 효율적 치료를 위해 2017년에‘치매 국가 책임제’를 발표함.

○ 더불어 최근에는 치매 치료제 개발을 세계적인 제약회사들이 순차적으로 포기하면서 치매 이전 단계인 인지장애를 치료하는 것에 더욱 관심이 증가하고 있음. 특히 연구자에 따라 차이를 보이기는 하지만, 경도인지장애로 진단받은 대상자 중 10~41%가 1년 안에 치매로 이환된다는 보고가 있음. 이는 결국 치매 이전 인지장애부터 선제적 관리와 치료가 필요함을 말해줌.

○ 2016년 전국치매역학조사 발표에 의하면, 우울증의 치매 위험도가 우울증 없는 사람보다 약 4.6배가량 높은 것으로 나타남. 우울증은 치매의 전조증상 혹은 치매 증상 중의 하나로 보고 노인에게 있어서 우울증과 치매는 서로 밀접한 연관성이 있는 것으로 보고됨. 치매 조기 진단에서 있어서 경도인지장애와 노인성 우울증 진단이 중요한 변수이며, 예후에도 영향을 미치는 것도 연구됨.

□ 조기 치매에 한의치료 효과 입증되었으나, 치매 환자 한의치료 소외

○ 노인의 노화와 관련한 신경질환인 치매와 인지장애의 경우는 인지기능의 점진적 악화를 예방하고, 행동 심리증상 및 정서적 지지와 함께 약물만이 아닌 대체 방안이 필요함.

○ 한의학적 치료는 이러한 인지기능의 개선 및 행동 심리증상과 환자에 대한 지지, 가족적 진료를 통한 노인성 치매 및 인지장애 증상 완화에 대한 일정한 효과를 보이고 있음. 이에 일선 한의사가 치매를 진료하고 관리하는 것은 국민에게 다양한 선택권을 보장하는 것과 더불어 더 많은 인지기능장애 환자 및 위험군에 혜택을 제공할 수 있을 것임.

□ 치매 환자의 한의약 치료 활성화 필요

○ 2011년 보건복지부 ‘치매 검진사업’ 『치매관리법』제정으로 제11조 제1항에 따라 치매 조기 발견을 위한 검진사업을 시행함에 있어서 치매 진단 후 치료 이용 의료기관으로 한의계 소외.

○ 치매 진단권은 인정하지만, 치료권이 없는 기이한 형태의 치매 보건소 사업. 보건소 검진 후 지정 협약 병, 의원 보장성 강화로 한의원 소외된 채로 거의 10년이 동안 고착됨.

○ 제도적 진입 근거 기반 부족이 이슈가 됨. 한의 의료기술이 인지장애 환자들에게 쉽게 접근할 수 있도록 첩약 급여화 및 보장성 강화를 위해 임상 현장이 반영된 근거 창출 시급함.

○ 경도인지장애, 치매 등의 인지장애와 노인에게 있어서 우울증과 치매 연관성이 높은 노년기 우울증에 대한 레지스트리 구축으로 한의 진료 경과에 따른 경도인지장애 혹은 우울증에서 치매로 가는 이환율과 치매에서 사망에 이르는 사망률 추산이 기존 DB와 비교 분석 가능함. 따라서 한의 진료의 근거를 가장 우선적으로 창출할 수 있는 인지장애 레지스트리 시스템 구축이 필수임.

○ 제2조(정의) 제2항. 법적으로 보장된 진단권이 있음에도 불구하고 치료권은 보장받지 못함. 한방신경정신과 전문의만 치매특별등급 소견서를 발급할 수 있고, 치매안심센터 협력 의사에서도 한의사는 제외됨.

□ 한의학에서는 건강노화를 위한 다양한 예방, 관리 방안을 제시

○ 한의학에서는 노화를 조절하기 위하여 침, 뜸 한약 등을 통해 인체의 근본 기운을 조절하고 노화 관련 질환 예방 및 치료 효과가 보고 되고 있음.
- 나이가 듦에 따라서 호르몬 분비, 수면 패턴, 체온유지, 혈압 등의 생체리듬에 변화가 유발. 이는 노인연관 질환을 유발하고 건강노화를 저해하는 요인으로 작용
- <동의보감> 신형문(身形門)에는 ‘사시절의(四時節宣)’, ‘연제법(煉臍法)’, ‘훈제비방(熏臍秘方)’, ‘구제법(灸臍法)’, ‘태식(胎息)’ 등의 자연과 자신의 생체리듬에 맞게 생활하여 건강하게 늙고 건강노화를 위한 다양한 방안에 대하여 제시

□ 지자체 중심 노인 인지장애 관련 보건소 한의치매사업 진행

○ 2016년 서울시 한의사회는 시예산 5억 원을 확보하여 시범사업을 진행하며 총 146개 한의원이 참여해 4주/8주 프로그램을 진행했음. 그 결과 만족도는 10점 만점의 총 9.02로 나타남. 이는 치맥 국가 책임제도와 함께 한의학의 영역을 더 확대할 수 있는 근거를 마련한 것으로 사료됨, 그 외 부산광역시, 경기도 등 지자체 중심의 보건소 한의치매사업 활발히 진행 중임.

○ 문재인 정부의 국정운영 5개년 계획 중 정신건강 분야 정책이 포함되어 있음. 이에 따라 정신건강 증진 체계 강화, 치매 국가책임제 내용을 포함한 국정과제 전략에 한의치매 진료 지침의 연계를 강화함(표 1).

| **세부이행체계** | **내용** |
| --- | --- |
| 정신건강증진  체계 강화 | -정신건강 관련 서비스 전달체계 개편 및 전문인력 충원과 근무 조건 개선  -자살 예방 및 생명 존중 문화 확산(정신건강서비스 이용률 제고 15% --> 20%) |
| 치매 국가 책임제 | -2017년부터 전국 252개 치매안심센터 확충 및 치매 안심병원 확충 추진  -2018년부터 중증 치매 환자 본인 부담률 인하 및 고비용 진단검사 급여화  -장기요양 치매 수급자 본인 부담 경감 확대 |

□ 노인 인지장애 관련 연구 현황

(1) 양방에서 노인성 치매를 포함 인지장애 관련 코호트 사업 및 병원 코호트(CREDOS) 현황

○ 현재 노인성 치매 연구에 대한 코호트는 2006년 이후 추적조사를 지속하고 있으며 현재 중앙치매센터에서 제공하는 치매 유병현황자료에 따르면 우리나라의 치매환자수는 약 84만 명으로 추산하고 있으며 2030년과 2050년에는 각각 127만 명, 271만 명까지 증가할 것으로 전망함

○ 건강 보험심사평가원의 2009년부터 2019년까지 총 11년간 전 국민 진료내용을 바탕으로 한 치매 질환 진료 현황 분석에 따르면 치매 질환 수진자 수와 진료 건수는 각각 3.6배, 5.5배 증가했으며, 진료비 증가율은 7.3배로 전체 진료비 증가율 3.4배보다 큰 폭으로 증가함

○ 또한 치매 질환 중 알츠하이머 치매는 2018년 전체 질병 중 요양급여비용 총액 1위를 차지하며 이는 2위 질환인 뇌경색 중 진료비의 약 1.4배에 달하는 수치임. 더불어 건강보험정책연구에 따르면 지역사회에서의 '건강 노화 실현(Healthy Aging in Place)'을 위한 정책개발의 근거 제공 목적으로 진행 중인 '장기 요양 노인 코호트 구축 연구'의 세 번째 과제를 시작함

(2) 노인 인지장애 관련 한의약 치료기술 및 지식재산권 현황

○ 한의학에서는 신경인지장애에서 나타나는 기억력 저하 및 인지기능장애, 정서행동문제, 인격 변화의 임상 증후를 치매(癡呆), 매병(呆病), 건망(健忘), 전광(癲狂), 허로(虛勞) 등의 범주에서 다루고 있음

○ 치매 한의변증평가도구는 현재 객관화와 표준화가 아직 확보되지 않아 임상에서 표준화된 진단도구로써 활용하기에 무리가 있었음. 이에 이고은 등은 문헌 검토 및 전문가 의견 조사를 기초로 인지장애 임상 증후를 기허(氣虛), 음허(陰虛), 화열(火熱), 담음(痰飮) 등 4개의 변증으로 평가할 수 있는 척도를 마련하고 임상연구를 통해 인지장애 변증평가도구(Pattern Identifications Tool for Cognitive Disorders, 이하 PIT-C) Ver. 2.1을 만들었고 이지윤 등이 임상연구를 통해 인지장애 변증도구 Ver. 2.1의 신뢰도와 타당도를 확보하였음.

(3) 노인 인지장애 관련 한의약 연구 현황

○ 경도인지장애 환자를 대상으로 침 시술을 진행한 결과 fMRI의 뇌 활성 변화, 임상적인 인지기능 지표 등의 변화를 관찰함. 그 결과 20여 개의 뇌 영역에서 활성의 변화가 측정됨 또한 고령 환자군에서 CDR (Clinical dementia rating scale score), MMSE(Mini-Mental state examination score)는 유의미하게 호전됨.

○ 기억감퇴형 경도인지장애에 대한 침치료 임상연구에 대한 메타분석 결과 578명이 참여한 5개의 임상연구가 분석되었으며, 침치료 군이 뇌혈류순환제의 일종인 nimodipine을 투여받은 군보다 임상적 효과가 높은 것으로 나타남.

○ 한약은 경도인지기능장애에 대한 약물치료 중 부작용이 적고 효과가 뛰어난 선택지 중 하나로 알려져 있음.

○ 기억력감퇴형 MCI 환자에 대한 Shenwu 캡슐 임상연구 결과 Donepezil 투여군과 최대 48주간 동등한 수준의 기억력 감퇴 방지 효과를 나타내었으며, 위장관계 및 정신신경계 부작용은 도네페질에 비하여 한약물 투여군이 적게 관찰되었음이 보고됨.

□ 한의학적 치료는 이러한 인지기능의 개선 및 행동 심리증상과 환자에 대한 지지, 가족적 진료를 통한 노인성 치매 및 인지장애 증상 완화에 대한 일정한 효과를 보이고 있음. 이에 일선 한의사가 치매를 진료하고 관리하는 것은 국민에게 다양한 선택권을 보장하는 것과 더불어 더 많은 인지기능장애 환자 및 위험군에 혜택을 제공할 수 있을 것임. 그럼에도 불구하고 현재까지 이런 치매 질환에 대한 자료를 수집하고 분석하여 Data를 구축한 실례는 한의에서는 존재하지 않은 상황임.

**3. 연구 목적**

□ 연구의 최종 목표

○ 본 연구는 인지장애(경도인지장애, 알츠하이머병, 혈관성치매) 환자에 대한 한의 진단, 치료, 관리, 예방 자료 축적을 위한 한의 레지스트리를 구축하고 인지기능에 영향을 미치는 데이터를 수집하고자 함.

**4. 연구 기관 및 주소**

**1) 주관기관 및 실시기관**

**(1) 주관기관**

| **기관명** | **주소** | **연구책임자** | **연락처** |
| --- | --- | --- | --- |
| 원광대학교  장흥통합의료병원 | 전남 장흥군 안양면 로하스로 121 | 강형원 | 061-860-7777 |

**(2) 실시기관**

| **기관명** | **주소** | **연구책임자** | **연락처** |
| --- | --- | --- | --- |
| 원광대학교  장흥통합의료병원 | 전남 장흥군 안양면 로하스로 121 | 강형원 | 061-860-7777 |
| 원광대학교 한방병원 | 전북 익산시 무왕로 895 | 임정태 | 063-850-6914 |
| 대전대학교 대전한방병원 | 대전광역시 서구 대덕대로 176번길 75 | 정인철 | 042-470-9129 |

**2) 주관기관 및 실시기관 인지장애 관련 통계**

**(1) 장흥통합의료병원**

- 원광대 장흥통합의료병원 2020년 전체 환자 수는 15,153명, 60세 이상 환자 수는 9,598명(전체 환자의 63.3%), 경도인지장애, 치매 환자 수는 19명(전체 환자의 0.1%)에 해당함.

- 2021년 전체 환자 수는 25,508명, 60세 이상 환자 수는 8,264명(전체 환자의 55.4%), 경도인지장애, 치매 환자 수는 17명(전체 환자의 0.1%)에 해당함.

- 2022년 전체 환자 수는 27,623명, 60세 이상 환자 수는 12,834명(전체 환자의 56.7%), 경도인지장애, 치매 환자 수는 22명(전체 환자의 0.2%)에 해당함.

- 전체적으로 60세 이상 환자 수가 증가하는 추세임.

**(2) 원광대학교 한방병원**

- 원광대학교 한방병원 2020년 전체 환자 수는 26,476명, 60세 이상 환자 수는 8,601명(전체 환자의 32.4%), 경도인지장애, 치매 환자 수는 61명(전체 환자의 0.2%)에 해당함.

- 2021년 전체 환자 수는 25,508명, 60세 이상 환자 수는 8,412명(전체 환자의 32.9%), 경도인지장애, 치매 환자 수는 41명(전체 환자의 0.2%)에 해당함.

- 2022년 전체 환자 수는 27,623명, 60세 이상 환자 수는 10,230명(전체 환자의 37%), 경도인지장애, 치매 환자 수는 49명(전체 환자의 0.2%)에 해당함.

- 전체적으로 60세 이상 환자 수가 증가하는 추세이며, 인지장애 환자군의 실 인원수는 적으나 1년간 지속적으로 내원하고 있음을 확인함.

**(3) 대전대학교 대전한방병원**

- 대전대학교 대전한방병원 2020년 전체 환자 수는 16,646명, 60세 이상 환자 수는 4,757명(전체 환자의 28.5%), 경도인지장애, 치매 환자 수는 70명(전체 환자의 0.4%)에 해당함.

- 2021년 전체 환자 수는 16,046명, 60세 이상 환자 수는 4,968명(전체 환자의 30.9%), 경도인지장애, 치매 환자 수는 32명(전체 환자의 0.2%)에 해당함.

- 2022년 전체 환자 수는 14,483명, 60세 이상 환자 수는 4,721명(전체 환자의 32.5%), 경도인지장애, 치매 환자 수는 52명(전체 환자의 0.4%)에 해당함.

- 전체적으로 전체 환자 수 대비 60세 이상 환자 수가 증가하는 추세임.

| **연도** | **구분** | **장흥통합**  **의료병원** | **원광대 한방병원** | **대전대**  **대전 한방병원** |
| --- | --- | --- | --- | --- |
| 2020 | 전체 환자 수 | 15,153 | 26,476 | 16,646 |
|  | 60세 이상 환자 수 | 9,598 | 8,601 | 4,757 |
|  | 치매/경도인지장애 총 내원 환자 수* | 61 | 1,264 | 432* |
|  | 치매/경도인지장애 실인원수 | 19 | 61 | 70 |
| 2021 | 전체 환자 수 | 14,908 | 25,508 | 16,046 |
|  | 60세 이상 환자 수 | 8,264 | 8,412 | 4,968 |
|  | 치매/경도인지장애 총 내원 환자 수* | 58 | 517 | 248* |
|  | 치매/경도인지장애 실인원수 | 17 | 41 | 32 |
| 2022 | 전체 환자 수 | 22,619 | 27,623 | 14,483 |
|  | 60세 이상 환자 수 | 12,834 | 10,230 | 3,240 |
|  | 치매/경도인지장애 총 내원 환자 수* | 92 | 925 | 472* |
|  | 치매/경도인지장애 실인원수 | 22 | 49 | 52 |
| *주 상병인 환자의 경우에만 해당 | | | | |

**5. 기관별 연구책임자**

| **기관명** | **주소** | **연구책임자** | **연락처** |
| --- | --- | --- | --- |
| 원광대학교  장흥통합의료병원 | 전남 장흥군 안양면 로하스로 121 | 강형원 | 061-860-7777 |
| 원광대학교 한방병원 | 전북 익산시 무왕로 895 | 임정태 | 063-850-6914 |
| 대전대학교 대전한방병원 | 대전광역시 서구 대덕대로 176번길 75 | 정인철 | 042-470-9129 |

**6. 연구 기간**

○ 승인일로부터 ~ 2029년 12월 31일

**7. 연구 대상자**

**1) 선정기준**

① 만 55세 이상 85세 이하 성인 남녀

② 정신질환의 진단 및 통계 편람 제 5판 (DSM-5) 기준에 따라 알츠하이머병으로 인한 주요신경인지장애, 혈관성질환으로 인한 주요신경인지장애, 경도신경인지장애로 진단된 자

③ 자의 또는 법정대리인의 의사로 참여를 결정하고 동의서에 서명한 자

**2) 제외기준**

① 알츠하이머형, 혈관성치매 이외의 원인으로 인한 치매, 즉 파킨슨병, 헌팅턴 병, 전두측두형 치매, 크로이츠펠트 야곱병 등의 퇴행성 뇌질환을 갖고 있는 자

② 치매를 일으키는 전신적 상태 즉, 갑상선 기능저하증, 비타민 B12 또는 엽산결핍, 나이아신 결핍, 과칼륨혈증, 신경매독, 인간 면역결핍 바이러스 병 등을 갖고 있는 자

③ DSM-5에 의해 진단된 정신병적 장애 및 물질관련장애, 즉 조현병, 망상장애, 양극성장애, 알코올 혹은 물질남용장애 등 주요 정신과적 장애 과거력이 있는 자

④ 뇌전증, 국소 뇌손상, 두부외상 등 신경과적 장애의 과거력이 있는 자

⑤ 식이요법 또는 약물치료 등으로 조절되지 않는 위장관, 내분비 및 심혈관계 질환을 가진 자

⑥ 심각하게 불안정한 의학적 상태인 자(임상실험실 검사, 심전도검사, Chest x-ray, 활력징후 등의 결과를 바탕으로 한 담당의사의 판단에 의함)

⑦ 연구자의 판단에 따라 본 임상연구에 참가가 적합하지 않은 자

**3) 중도 탈락 기준**

① 임상연구대상자가 임상연구 참가 동의를 철회한 경우

② 연구대상자와 연락이 안되는 경우와 같이 추적검사가 불가능한 경우

③ 기타 담당자의 판단에 의해 연구의 진행이 적합하지 못한다고 판단되는 경우

**8. 연구 대상자 수와 산출 근거**

**1) 조사 모집단 및 목표 등록 인원**

① 모집 기관: 원광대학교 장흥통합의료병원, 원광대학교 한방병원, 대전대학교 대전한방병원

② 모집 대상: 만 55세 이상 85세 이하 성인 남녀 중 정신질환의 진단 및 통계 편람 제 5판 (DSM-5) 기준에 따라 알츠하이머병으로 인한 주요신경인지장애, 혈관성질환으로 인한 주요신경인지장애, 경도신경인지장애로 진단된 자

③ 목표 등록 인원 : 총 300명, 2024년부터 2028년 사이에 신규 환자를 경쟁적으로 모집하며 2028년 12월까지 원광대학교 한방병원, 원광대학교 장흥통합의료병원, 대전대학교 대전한방병원에서 경쟁적 방식으로 총 300명을 모집함. 기관별 모집인원 약 100명이나, 경쟁적 모집으로 변동될 수 있음. 2024년-2029년 사이에 년 1회 방문(최소1년 ~최장6년 관찰), 1200 person-year에서 탈락률 30% 고려하여, 최종 800 person-year 이상

2) 표본 추출 방법

○ 본 연구의 목적은 인지장애의 예후에 영향을 미치는 요인을 파악하기 위한 레지스트리 연구로, 미리 정해진 통계적 가설을 검정할 필요가 없으므로 사전 표본 크기 계산이 필요하지 않음. 그러나 연구비와 연구 상황을 고려하여 표본 추출이 현실적으로 가능한 범위내에서 표본수를 정하고자 함.

○ 모집단 대표성에 대한 한계는 있지만 현실적인 적용 가능성을 고려, 원광대학교 한방병원, 원광대학교 장흥통합의료병원, 대전대학교 대전한방병원에서 만 55세 이상 85세 이하 성인 남녀 중 정신질환의 진단 및 통계 편람 제 5판 (DSM-5) 기준에 따라 알츠하이머병으로 인한 주요신경인지장애, 혈관성질환으로 인한 주요신경인지장애, 경도신경인지장애로 진단된 자를 조사대상자를 선정하고자 함.

○ 60명씩 5년에 걸쳐 신규환자를 추가적으로 모집하여 총 300명을 대상으로 조사하는 방안이며 연도별 사망자 발생 및 기타 사유에 따른 레지스트리 손실이 있을 수 있으나, 유지율 최소한 70% 이상 유지하는 것을 목표로 함.

○ 3개 기관의 3년간 치매/경도인지장애 실인원수를 합산하여 1년 평균을 내면 121명임. 그 중 50% 정도 동의하고 참여할 것으로 예측하면 연 60명, 5년간 300명 등록을 목표로 함. 총 누적 1200 person-year에서 탈락률 30% 고려하여, 최종 840 person-year 이상을 목표로 함.

○ 한의치료를 받지 않아도 연구에 참여할 수 있음. 연구기간 내 한 번도 한의치료를 받지 않아도 됨.

**9. 연구 방법 및 절차**

**1) 연구 방법**

□ 레지스트리 구축을 통한 환자 등록

- 레지스트리 연구는 특정 질병 또는 특정 인자에 노출된 모집단의 결과를 평가하기 위해 균일한 데이터를 지속적으로 수집하고 의미 있는 결과를 도출할 수 있도록 조직화된 시스템을 통해 데이터를 수집하는 관찰연구임.

- 본 연구는 인지장애(경도인지장애, 알츠하이머병, 혈관성치매) 환자를 대상으로 레지스트리를 구축하고, 이후 매년 임상 적용 평가를 통해 인지기능에 영향을 미치는 데이터를 수집하고자 함.

- 자의 또는 법정 대리인의 의사에 의해 임상연구 동의서에 서명한 피험자를 대상으로 선정기준 및 제외기준을 검토하여 임상연구에 적합하다고 판단이 되면 레지스트리 등록을 실시함. 원광대학교 한방병원, 장흥통합의료병원, 대전대학교 대전한방병원에서 각각 경쟁적으로 환자등록을 시행할 것임.

- 성공적인 등록연구 수행을 위하여 각 기관에서는 레지스트리연구 담당 연구코디네이터를 채용할 것이며 의사소통체계 확보를 통하여 각 기관의 레지스트리연구 대상자 모집정도를 수시로 파악할 것임.

□ 레지스트리 구축 및 문제 대처 전략

- 연구 대상자는 연 1회 의료기관을 방문하여 SOP에 따라 설문 검사와 신체 검진을 받고 다음 방문 교육을 실시함.

- 레지스트리 연구에서 나타나는 가장 큰 문제점은 대상자 추적조사의 어려움임. 탈락율을 최소화하기 위하여 연 1회 이상 생활습관 등의 건강과 인지장애 관련 자료를 배포하여 레지스트리 연구 참여 대상을 인지시키고, 연구코디네이터가 유무선 연락을 취해 건강관리 상태를 확인할 예정임.

□ 생체지표 조사 전략

- 레지스트리 구축 후 연구코디네이터 및 연구원을 대상으로 생체지표 조사를 위한 교육을 실시할 예정임.

- 심전도, 흉부 X-ray, 혈액샘플 채취, 소변검사는 원광대학교 한방병원, 원광대학교 장흥통합의료병원, 대전대학교 대전한방병원의 진료 시스템을 따라서 소속 연구원을 통해 진행할 것이며, 심전도, 흉부 X-ray, 소변검사는 원내에서 실시하며 채취한 혈액 및 소변은 24시간 이내에 분석항목에 대해서 분석 진행할 예정임.

**2) 연구대상자 동의 및 모집**

**1] 연구대상자 동의서 및 모집공고**

□ 임상연구 참가 동의서, 개인정보이용 동의서

- 참여 동의를 얻기 전, 연구자는 IRB 승인을 받은 연구 설명문과 동의서를 참여를 원하는 대상자에게 충분히 설명한다. 본 연구의 목적과 연구의 활용, 비밀 유지에 대한 내용, 실시기관에서 수집한 데이터는 분석을 위해 공동연구기관에 이양될 수 있음, 대상자가 원할 경우 언제든지 연구 참여를 철회할 수 있음을 설명하고 대상자의 질의에 응답한다. 대상자의 서면 동의를 얻은 후 동의서 사본을 대상자에게 제공한다.

- 선정·제외기준을 통해 주요신경인지장애로 진단된 자는 취약한 대상자로 분류해 대상자와 법정대리인의 동의를 취득한다. 경도신경인지장애로 진단된 자는 본인 동의만 취득하며, 본 서명은 전체 연구기간의 참여에 대한 동의를 의미한다. 단, 대상자가 재동의 작성을 요청하는 경우 등 추가 동의를 취득하여 진행할 수 있다. 만약, 대상자의 추적 방문 시 최초 동의 시점 이후 대상자의 의학적 상태가 변화되었다고 연구자가 판단하였을 경우에는 취약한 대상자로 분류하여 대상자와 법정대리인의 추가 동의를 취득한다.

- 법정대리인의 동의능력은 연구한의사의 판단하에 필요한 경우에는 외래 진료 등을 통하여 평가를 진행한다. 법정대리인의 동의 능력이 저하되어 있으면 해당 환자는 등록을 하지 않거나 다른 법정대리인의 동의를 받도록 한다.

- 법정대리인 동의의 경우 법정대리인이 작성해야하며, 법정대리인에 대한 신분에 대해 증명할 수 있는 문서를 확인하고 보관한다. (가족관계증명서 등)

□ 연구 대상자 모집 방법

- 본 연구에 대한 모집 광고문을 연구 대상자 모집 기관의 원내 게시판에 부착하며 홈페이지상에 팝업창을 통해 게시한다. 추가로 익산시 관내 보건소, 익산시 한방의료기관에도 부착하며 지역신문 광고, 대중교통 광고, 허가받은 환자 모집 사이트 등을 통해서도 모집한다. 모든 연구 대상자 모집은 임정태 책임연구자가 담당한다.

- 알츠하이머병으로 인한 주요신경인지장애, 혈관성질환으로 인한 주요신경인지장애, 경도신경인지장애로 진단되는 환자에게 본 연구에 대해 설명한다. 위 과정에서 자발적으로 연구에 동의하는 환자들에게 연구에 관해 상세히 설명하고 서면 설명문을 제공한다. 최종적으로 연구에 참여하기로 결정하고 서면 동의서를 제출한 환자들에 대해 선정/제외 기준 적합성을 평가하고, 연구 참여에 적합하다고 판단되는 환자들을 최종 등록한다.

□ 자발적 연구 참여와 동의철회

- 연구대상자는 본 연구에 참여하지 않을 자유가 있으며 본 연구에 참여하지 않아도 어떠한 불이익이 없음을 알린다. 또한, 연구대상자는 연구에 참여한 언제든지 동의를 철회하고 도중에 그만 둘 수 있으며, 만일 연구에 참여하는 것을 그만두고 싶다면 담당 연구원이나 연구책임자에게 즉시 연락하도록 알린다. 단, 연구 참여에 대한 동의를 철회시에도 그때까지 수집된 자료는 임상연구와 관련한 목적에 한해 열람, 처리 및 활용될 수 있다.

**2] 연구 대상자 모집 및 등록 방법**

- 동의를 받는 순서에 따라 다음의 스크리닝 번호를 부여한다. 피험자 스크리닝 번호는 다음 방법에 따라 기록한다.

- 실시기관 코드 : 원광대학교 장흥통합의료병원(WJH), 원광대학교 한방병원(WKH), 대전대학교 대전한방병원(DJH)

- 실시기관 코드-실시년도-Screening의 첫 글자-등록된 순서

(ex : WKH-2023-S-015: 원광대학교 한방병원 2023년도 15번째 스크리닝 환자)

- 피험자 식별코드는 다음 방법에 따라 기록한다.

- 실시기관 코드 : 원광대학교 장흥통합의료병원(WJH), 원광대학교 한방병원(WKH), 대전대학교 대전한방병원(DJH)

- 실시기관 코드-실시년도-Enrollment의 첫글자-등록된 순서

(ex : WKH-2023-E-015: 원광대학교 한방병원 2023년도 15번째 등록된 환자)

- 대상자의 이름은 이니셜로 표기한다

| **대상자 식별코드** | **대상자 이니셜** |
| --- | --- |
| \|  \|  \|  \| \| --- \| --- \| --- \|   **– 202_ - S -**   \|  \|  \|  \| \| --- \| --- \| --- \| | \|  \|  \|  \| \| --- \| --- \| --- \| |
| \|  \|  \|  \| \| --- \| --- \| --- \|   **– 202_ - E -**   \|  \|  \|  \| \| --- \| --- \| --- \| |  |
| □ NA (Screening Fail) |  |

**3) 연구절차**

**1] 방문기간 및 간격**

- 연구등록 시점부터 2029년 12월까지 1년마다 1회 방문함. 따라서 7년간의 본 연구의 2년차인 2024년에 등록된 환자는 6년간 관찰하게 되고, 6년차에 등록된 환자는 2년간 관찰하게 됨.

| 스크리닝 | 연구 참여 동의 | - 연구대상자 및 법정대리인 동의서 |
| --- | --- | --- |
|  | 스크리닝 조사 | - 인구학적 조사, 병력 및 약물투여력 조사 - 활력징후 및 신장, 몸무게 - 실험실 검사 - 환자 선정·제외를 위한 설문평가 |
| Visit  1 | ^기저조사^ | - 기초설문 (인지장애 변증도구) - 장기요양서비스 이용 현황 - 보호자 설문 평가 - 환자 설문 평가 - 기기 검사 - 질병력, 치료력 및 병용약물 확인 - 방문일정 교육 |
| Visit  2~6  (공통) | ^추적조사^ | - 실험실 검사 - 활력징후 및 신장, 몸무게 - 장기요양서비스 이용 현황 - 보호자 설문 평가 - 환자 설문 평가 - 기기 검사 - 질병력, 치료력 및 병용약물 확인 - 방문일정 교육 |

**2] 조사 내용**

(1) 스크리닝 조사

① 인구학적 특성(흡연력, 음주력, 질환의 가족력, 학력, 문자해독, 직업, 결혼여부, 운전여부, 전화사용여부, 보호자 정보, 의료보험형태, 간병비용, 병원비 부담 등 & 보호자의 성별 및 나이, 주소, 전화번호, 직업, 과거력 및 현병력, 부담감, 환자와 관계, 동거여부, 돌보는 시간, 월 평균 수입, 환자 월 지출액, 인지치료 여부, 인지장애 관련 보험 여부 등), 병력 및 약물투여력(최근 3년 이내의 과거 병력 및 현재 병력. 치매·뇌졸중 가족력, 기왕력, 생활습관, 약물복용력), 활력징후 및 신장과 몸무게를 조사함.

② 실험실검사: 임상병리검사 항목은 아래와 같으며 필요에 따라 다른 추가 검사 수행할 수 있음. (*표시는 스크리닝에만 수행)

□ 원광대학교 장흥통합의료병원, 원광대학교 한방병원 (원내 검사실에서 수행함)

- 혈액학적 검사 : WBC, RBC, Hemoglobin, Hematocrit, Platelet, MCV, MCH, MCHC, Fibrinogen*

- 혈액화학적 검사 : Glucose, BUN, Creatinine, AST, ALT, ALP, γ-GTP, Total Bilirubin, Albumin, Total Protein, Total Cholesterol, Triglyceride, HDL/LDL Cholesterol, TSH, Free T4, CRP, Vitamin B12*, Folate*, Homocysteine*, TPHA* , VDRL* (TPHA와 VDRL검사가 불가능한 경우 매독 배제진단을 위해 기관 현황에 따라 syphillis 검사를 통해 배제진단을 하는 것을 허용함)

- 소변검사 : Specific gravity, Nitrite, pH, Protein, Glucose, Ketone, Urobilinogen, Bilirubin, WBC, RBC

- 심전도, 흉부 X-ray

□ 대전대학교 대전한방병원 (원내 검사실에서 수행함)

- 위 항목에서 VDRL, TPHA를 syphillis 검사로 대체함

③ 환자 설문 평가 : 인지기능 평가(K-MMSE2, MoCA-K, GDS), 우울(S-GDps)

(2) 기저 조사 (방문 1)

① 기초 설문 (인지장애 변증도구)

② 보호자 설문 평가

가. 환자 관련 평가 : 환자 일상생활능력평가(K-BADL, S-IADL), 이상행동평가(NPI-Q)

나. 보호자 관련 평가 : 주보호자 부양부담감

③ 환자 설문 평가 : 삶의 질(GQOL-D, EQ-5D-5L, EQ-VAS), 혈쇠척도, 핵심칠정척도 단축형

④ 장기요양서비스 이용 현황: 방문요양, 방문목욕, 방문간호, 주간 또는 야간보호, 단기보호, 시설서비스

⑤ 질병력, 치료력 및 병용약물 변동사항

⑥ 기기 검사

가. 필수: HRV

니. 환자상태에 따라 선택: QEEG, fNIRS

⑦ 다음 방문일정 교육

(3) 추적조사 (방문 2 ~ 방문 6)

-기저조사 후 1년마다 추적조사한다.

-질병력, 치료력 및 병용약물 변동사항, 활력징후 및 신장, 몸무게, 실험실검사, 보호자 설문평가, 환자 설문 평가, 장기요양서비스 이용 현황, 기기 검사, 다음 방문일정 교육

임상연구 진행 일정표(Study Flow Chart)

| **방문 (Visit)**^1)^ | | **screening** | **1** | **2** | **3** | **4** | **5** | **6** |
| --- | --- | --- | --- | --- | --- | --- | --- | --- |
| **Year** | |  | **0** | **1**  **(±30일)** | **2**  **(±30일)** | **3**  **(±30일)** | **4**  **(±30일)** | **5**  **(±30일)** |
| 대상자 및 법정대리인 동의서 취득 | | ● |  |  |  |  |  |  |
| 선정/제외기준 확인 | | ● |  |  |  |  |  |  |
| 스크리닝 번호 부여 | | ● |  |  |  |  |  |  |
| 인구학적 조사^2)^ | | ● |  |  |  |  |  |  |
| 병력 및 약물투여력 조사^3)^ | | ● |  |  |  |  |  |  |
| 활력징후 및 신장, 몸무게^4)^ | | ● |  | ● | ● | ● | ● | ● |
| 실험실검사^5)^ | | ● |  | ● | ● | ● | ● | ● |
| 장기요양서비스 이용 현황 | |  | ● | ● | ● | ● | ● | ● |
| 인지장애 변증도구 | |  | ● |  |  |  |  |  |
| 보호자 | 주보호자 부양부담감 |  | ● | ● | ● | ● | ● | ● |
| 환자 상태  (보호자가  체크) | K-BADL |  | ● | ● | ● | ● | ● | ● |
|  | S-IADL |  | ● | ● | ● | ● | ● | ● |
|  | NPI-Q |  | ● | ● | ● | ● | ● | ● |
| 환자 상태 | K-MMSE2 | ● |  | ● | ● | ● | ● | ● |
|  | MoCA-K | ● |  | ● | ● | ● | ● | ● |
|  | GDS | ● |  | ● | ● | ● | ● | ● |
|  | S-GDpS | ● |  | ● | ● | ● | ● | ● |
|  | 핵심칠정척도 단축형 |  | ● | ● | ● | ● | ● | ● |
|  | 혈쇠척도 |  | ● | ● | ● | ● | ● | ● |
|  | EQ-5D-5L |  | ● | ● | ● | ● | ● | ● |
|  | EQ-VAS |  | ● | ● | ● | ● | ● | ● |
|  | GQOL-D |  | ● | ● | ● | ● | ● | ● |
| 기기 검사  (필수) | HRV |  | ● | ● | ● | ● | ● | ● |
| (환자상태에 따라 선택) | QEEG |  | ● | ● | ● | ● | ● | ● |
|  | fNIRS |  | ● | ● | ● | ● | ● | ● |
| 질병력, 치료력 및 병용약물 변화 확인 | |  | ● | ● | ● | ● | ● | ● |
| 방문일정 교육 | |  | ● | ● | ● | ● | ● | ● |

1) Visit 1은 스크리닝 기준으로 10일 이내에 이루어져야 하며 당일 검사 결과가 나온 경우 1차 방문 조사를 수행할 수 있다. Visit 2~6은 Visit 1로부터 1년으로 하되 예정일 기준으로 ±30일 이내에 이루어져야 한다.

2) 인구학적 조사: 인구학적 정보 (대상자 이니셜, 성별, 생년월일, 연락처, 주소, 학력, 직업 등)을 조사한다.

3) 병력 및 약물 투여력 조사: 최근 3년 이내의 과거 병력 및 현재 병력을 조사하고 약물 투여력을 조사한다.

4) 활력징후 및 신장, 몸무게: 체온, 혈압 (이완기/수축기)과 맥박수를 측정한다.

5) 실험실검사: 임상병리검사 항목은 아래와 같으며 필요에 따라 다른 추가 검사 수행할 수 있다. (*표시는 스크리닝에만 수행)

□ 원광대학교 장흥통합의료병원, 원광대학교 한방병원 (원내 검사실에서 수행함)

- 혈액학적 검사 : WBC, RBC, Hemoglobin, Hematocrit, Platelet, MCV, MCH, MCHC, Fibrinogen*

- 혈액화학적 검사 : Glucose, BUN, Creatinine, AST, ALT, ALP, γ-GTP, Total Bilirubin, Albumin, Total Protein, Total Cholesterol, Triglyceride, HDL/LDL Cholesterol, TSH, Free T4, CRP, Vitamin B12*, Folate*, VDRL*, Homocysteine*, TPHA* (TPHA와 VDRL검사가 불가능한 경우 매독 배제진단을 위해 기관 현황에 따라 syphillis 검사를 통해 배제진단을 하는 것을 허용함)

- 소변검사 : Specific gravity, Nitrite, pH, Protein, Glucose, Ketone, Urobilinogen, Bilirubin, WBC, RBC

- 심전도, 흉부 X-ray

□ 대전대학교 대전한방병원 (원내 검사실에서 수행함)

- 위 항목에서 VDRL, TPHA를 syphillis 검사로 대체함

○ 설문평가 조사 항목 상세내용

(1) 일상생활능력평가

① 노인 일상활동평가 복합(Seoul-Instrumental Activities of Daily Living, S-IADL)

- S-IADL은 우리나라의 문화적 특성에 맞도록 문항을 제작하고 표준화 연구를 마쳤다. 0-3점 척도로 이루어진 15문항으로 구성되어 있다. 점수범위는 0-45점까지로 원점수의 합으로 산출되고, 점수가 높을수록 일상생활에 장애가 많음을 의미한다. 특히 이 도구는 IADL을 현재실행능력과 잠재능력으로 구분하여 평가한다. "현재실행능력"은 현재 환자가 독립적으로 IADL을 수행할 수 있는지를 평가하는 것이다. 반면 "잠재능력"은 현재 IADL을 독립적으로 수행하지는 않더라도, 환자의 잠재적인 능력을 고려하여 실행할 수 있는지의 여부를 평가한다. 이 도구는 정상인과 치매 환자집단이 절단점수 7.5점으로 구분(8점 이상이면 치매)될 수 있음을 보였고, 신뢰도와 타당도가 우수하였다.

② 한국어판 바델지수(Korean version of Barthel activities of daily living index, K-BADL)

- 기초적 일상생활 능력의 척도로 한국어판 바델지표(Korean version of Barthel activities of daily living index, K-BADL)이다. 총 20점 만점으로, 11~15점은 중등도의 장애, 10점 이하는 심각한 장애로 간주한다.

(2) 이상행동평가

① 신경정신행동검사-간편형(Brief Questionnnaire form of the Neuropsychiatric Inventory, NPI-Q)

- NPI-Q는 진료 현장에서 간편하게 사용하기 위하여 제작되었다. 12항목 모두 NPI와의 상관관계가 우수하였고(r=0.71-0.93), NPI는 15분 이상이 필요하지만 NPI-Q는 소요시간이 5분 이하인 장점이 있다. NPI와의 차이점은 다음과 같다. 첫째, 면담 형식이 아니고 보호자가 읽고 표시하는 설문지 형식이다. 둘째, 12가지 이상행동 각각에 대해 선별질문만 있고 세부질문이 없다. 셋째, 이상행동의 심한 정도와 보호자 고통 정도는 평가하나 빈도는 평가하지 않는다. 한국어로 번역된 NPI-Q를 사용한다.

(3) 삶의 질 평가(EQ-5D, EQ-VAS, GQOL-D)

① 건강관련 삶의 질 평가 척도 (EuroQoL-5 Dimension: 이하 EQ-5D-5L)

- EQ-5D는 1987년에 설치된 EuroQol 그룹에 의해 설치되고 연속적으로 개발되었다. Korean EuroQol-5 dimension(KEQ-5D)는 건강관련 삶의 질을 효용(utility)으로 측정하는 도구로 다차원적 선호도 근거 건강관련 삶의 질 측정도구(multidimensional preference based HRQOL measure)이다. 최근 EuroQol Group은 EQ-5D-3L의 단점을 보완하기 위해 EQ-5D-3L의 각 영역별 domain을 3개에서 5개로 널린 새로운 삶의 질 측정 도구를 발표하였다. EQ-5D-3L은 총 243개의 건강상태를 표현할 수 있었으나 EQ-5D-5L은 3,125(=55)개의 건강상태를 표현하기 되면서 EQ-5D-3L의 천장효과를 줄이고 신뢰도와 민감도를 증가시키면서 기술적 풍부함 또한 향상시킬 수 있을 것으로 기대된다. 실제로 국내 1개 병원의 암환자를 대상으로 EQ-5D-3L과 EQ-5D-5L 두 가지 도구를 비교한 연구에서 EQ-5D-5L은 EQ-5D-3L에 비하여 천장효과가 적고 타당도가 높았으며 검사-재검사 신뢰도는 유사하다는 연구 결과를 보고하였다. 현재 EuroQol Group에 의해 EQ-5D-5L의 한국어판 공식 버전이 제공되고 있다.

② 전반적 건강상태 점수 (EuroQoL Visual analogue scale:이하 EQ-VAS)

- EQ-VAS는 등급척도(rating scale) 중 하나로 20cm의 수직선 모양으로 나타낸 시각아날로그 척도이다. 상상할 수 있는 최고의 건강상태를 100점으로, 상상할 수 있는 최저의 건강상태를 0점으로 부여하고 주어진 건강상태에 대해 0점과 100점 사이의 점수를 부여하게 된다. 등급 척도로 얻은 점수는 건강결과의 순서를 분명하게 알려주며, 선호도에 대한 강도의 정보도 알려준다. 그러나 등급척도는 측정 비뚤림이 생기기 쉽다. 등급척도는 대체로 다른 도구의 보조적 사용이 권장되며(한국보건의료 연구원, 2013), EQ-VAS 역시 EQ-5D-5L 및 EQ-5D-3L 표준검사지에 보조적으로 포함되어 있다.

③ 치매노인의 삶의 질 척도(Geriatric Quality of Life-Dementia)

- ‘치매노인의 삶의 질’척도는 신체적 건강, 심리적 건강, 사회적 관계, 환경을 측정하는 13개 문항과 전반적인 건강 및 삶의 만족도를 측정하는 각 1개 문항 등 총 15개 문항으로 Likert 3점 척도로 구성되어 있다. 각 문항에 대한 반응의 단순 합으로 총점을 계산하며, 총점의 범위는 15-120점으로, 총점을 성별과 연령을 고려한 규준점수(T점수)로 환산하여 해석함. 규준점수가 높을수록 환자가 주관적으로 경험하는 삶의 질, 즉 만족도가 높다는 것을 의미한다. 특히 규준 점수 35T이하인 경우에는 삶의 질이 낮다는 것을 나타낸다.

(4) 간이정신상태검사 (Mini Mental Status Examination, MMSE)

- MMSE 은 가장 널리 사용하고 있는 선별검사의 하나이다. 이는 Folstein 등(1975)이 개발한 간단한 선별검사로서, 총점은 30점이고, 검사에 5-15분 정도 소요된다. 특히 학습 효과가 적어서 질병이 진행되는 동안 반복 측정함으로써 시간에 따른 변화를 살펴볼 수 있다는 장점이 있다(Foistein et al, 1975). 또한 중등도와 중증 치매 환자의 선별에 신뢰도와 타당도가 입증되어(Kaszniak et al., 1986) 우리나라에서 가장 널리 사용되는 검사법이다.

- 현재 국내에서는 강연욱 등(1997)이 번안한 K-MMSE와 권용철 등(1989)이 번안한 MMSE-K가 널리 시용되고 있다. 본 연구에서는 K-MMSE를 사용하며, K-MMSE는 총점이 30점이고 시간에 대한 지남력(5점), 장소에 대한 지남력(5점), 기억등록(3점), 주의집중 및 계산(5점), 기억회상(3점), 언어 능력(8점) 및 시공간 구성력(1점)으로 구성되었다. MMSE는 실시 방법이 까다롭지 않아서 간단한 교육을 받으면 쉽게 실시할 수 있다는 장점이 있지만, 교육수준과 연령, 문화와 언어의 차이에 많은 영향을 받고, 전두엽 기능을 평가하는 항목이 부족하여 전두측두치매나 혈관치매를 정확히 감별할 수 없으며, 난이도의 범위가 좁아 아주 경미하거나 심한 기억장애를 구별하지 못하는 단점이 있다.

- 일반적으로 총 23점을 인지기능장애의 평가기준점(절단점)으로 삼는다. 역학적 연구 결과들은 MMSE총점에 따라서 24~30점은 인지적손상 없음, 18~23점은 경도의 인지기능장애, 그리고 0~17점은 분명한 인지기능장애로 분류하고 있다.

(5) 한국판 몬트리올 인지평가(Montreal Cognitive Assessment-Korean, MoCA-K)

- MoCA-K는 MMSE 검사로 정상 소견을 보이는 경도인지장애를 선별하기 위하여 개발된 방법이다. 단기기억, 시공간 능력, 수행기능, 집중-작업기억, 언어, 지남력으로 구성되어 총 30점 만점으로 되어있다. 실행시간은 10분가량 소용되며 23점 이상이면 정상으로 간주하며 글을 읽거나 쓰는 능력이 없거나 서툰 경우에는 MoCA-K는 권장되지 않는다.

(6) 노인 우울증상 평가(S-GDpS(Short form Geriatric Depression Scale)

- S-GDpS(Short form Geriatric Depression Scale)은 노인 우울증상을 평가하기 위해 Cho 등이 한국판으로 개발한 노인우울척도(Geriatric Depression Scale, GDS)를 15문항으로 단축형을 사용하였다. 우울증 의심점수는 5점이다.

(7) 전반적 퇴화 척도(Global Deterioration Scale, GDS)

- GDS는 유럽 쪽에서 많이 시용되는 치매퇴화정도 척도로서 한국어로 번역하여 표준화 연구가 시행되었다. GDS 1은 인지장애가 없는 임상적으로 정상에 해당하고, GDS 2는 주관적 기억장애에 해당하며, 경도인지장애군은 GDS 3에 해당한다. 일부의 경도치매환자들이 GDS 3에 포함된다. GDS 4 부터는 확실히 치매라고 할 수있으며, GDS 4는 경도치매, GDS 5는 중등도 치매, GDS 6와 7은 심한 치매이다.

(8) 혈쇠척도

- 혈쇠척도는 한의사 치매진단 소견서 작성지침(장기요양 5등급) 중 포함된 한의평가도구로, 한의 병리적 노화척도이다. 노화에 대한 감각 및 신체증상으로 구성된 혈쇠척도는 총 9개 항목으로 없음이 0점, 각금 있음이 1점, 자주 있음이 2점으로 계산되며, 점수가 낮을수록 혈쇠척도가 양호한 것을 의미한다.

(9) 핵심칠정척도 단축형 (The Core Seven-Emotions Inventory Short Form: 이하 CSEI-s)

- 28문항으로 구성되어 희(喜) 4문항, 노(怒) 4문항, 사(思) 4문항, 우(憂) 4문항, 비(悲) 4문항, 공(恐) 4문항, 경(驚) 4문항이며, 각 문항별로 ‘전혀 그렇지 않다=1, 약간 그렇다=2, 웬만큼 그렇다=3, 꽤 그렇다=4, 매우 그렇다=5점’의 5점 리커트(Likert) 척도로 구성되어 있다.

- 본 척도는 T점수(평균 50점, 표준편차 10점)를 기준으로 하여 희(喜)를 제외한 노(怒), 사(思), 우(憂), 비(悲), 공(恐), 경(驚)은 감정별로 점수가 높을수록 고위험군에 속한다. 구체적인 절단점은 T점수 55∼60점 이하는 주의군, 61∼65점은 위험군, 66점 이상은 고위험군이다. 반면에 희(喜)의 경우는 낮을수록 위험군으로서 40∼45점 이하는 주의군, 35∼39점 이하는 위험군, 34점 이하는 고위험군이다. (단, 치매 중증도환자의 경우 감정카드로 대체할 수 있음. 더불어 치매 노인들의 정서측정은 인지능력의 손상에도 불구하고 MMSE-K점수가 10점 이상이면 스스로의 내적 상태를 표현하는데 큰 어려움이 없다는 것이 보고되고 있음.)

(10) 인지장애 변증도구

- 한국보건산업진흥원 지원 치매 한의표준진료지침 개발 과제(2016~2021) 중 수행된 인지장애 변증평가도구의 신뢰도와 타당도 평가 임상연구에서 신뢰도뿐만 아니라 타 인지장애 평가도 구들과의 상관성도 제시되었다. 이 도구는 최상위 변증유형을 결정하는 것뿐만 아니라 다른 변증유형의 경향성을 정량적으로 확인할 수 있기 때문에 한약, 침 치료의 주 처방 선택, 한약 수증가감, 병행 침 치료혈위 선택 및 치료 경과에 따른 평가에도 활용할 수 있다.

**3] 이상반응**

- 본 연구는 중재가 없는 레지스트리 연구로 예상되는 위험성이나 유해사례가 낮을 것으로 판단된다. 첫 방문 이후 추적 방문에서 새롭게 수집되는 모든 병력을 이상반응이 아닌 병력으로 수집하고, 대상자가 사망하였을 경우에만 이상반응으로 수집하여 기록한다. 본 연구는 중재가 없는 레지스트리 연구로 연구 참여로 인한 사망(연구와의 관련성이 있는 사망)은 없을 것으로 예상되나, 고령자를 대상으로 한 연구로 연구 진행 중에 사망하는 경우가 발생할 것으로 예상된다. 따라서, 기관별로 지속심의 시 전체 기관의 누적 사망 건을 모아서 함께 보고한다.

**10. 자료 수집 및 관리**

○ 자료수집 방법 및 데이터베이스 구축

- 본 연구의 제출된 종이 증례기록지를 기반으로 동일한 항목에 대해 한의약진흥원에서 관리하는 전자증례기록체계(e-CRF)를 개발하여 자료취합 및 입력을 수행하고, 이후 데이터 클리닝(data cleaning)등 절차를 거쳐 데이터베이스가 구축되고 분석될 계획임.

○ 의료기관내 의무기록·설문·전산자료 수집

- 연구 참여 의료기관에서의 의무기록·설문·전산자료 수집 절차는 다음과 같음.

1) 먼저 연구 참여 의료기관들의 기관생명윤리위원회(IRB)의 심의 및 승인을 받을 계획임.

2) 각 의료기관의 참여 연구진, 의무기록·설문·전산자료 조사 담당자와의 협의를 통해 연구 및 자료 수집 방법들을 확정함.

3) 선정기준에 따라 선정된 연구대상자들에 대하여 기관별, 환자별로 환자 등록번호를 부여함.

4) 환자등록번호를 기준으로 의무기록·설문·전산자료 조사를 완료한 근거문서 혹은 워크시트 원본은 각 의료기관에서 보관하고, 각 기관에서 연구담당자가 e-CRF를 원격 접속하여 증례기록서를 입력함.

○ 자료의 모니터링 및 질 관리

- 모니터링은 CRO (아리비앤씨)를 통해서 수행한다
- 모니터링과 관련한 제반 절차는 관련 규정 및 모니터링 계획서에 따라서 수행한다. 모니터링의 상세계획은 첨부한 모니터링 계획서(별첨01. 모니터링 계획서)를 따른다.

**11. 자료 분석 방법**

본 연구는 레지스트리 연구로, 미리 정해진 primary outcome은 없으나 다음의 분석방법을 따른다.

대상자 특성은 명목형 변수는 빈도(frequency)와 비율(ratio)로 제시하며, 연속형 변수는 평균과 표준편차 (mean, standard deviation) 으로 제시한다.

전체 환자 혹은 특정 하위집단에서의 연속변수의 변화는 정규성 검정 결과에 따라 paired t-test 혹은 Wilcoxon signed rank test를 사용한다. 명목형 변수의 변화는 Mcnemar’s test를 사용한다. 연속형 변수의 연관성은 Pearson’s 상관분석 (혹은 Spearman 상관분석)

특정 연속형 종속변수에 미치는 공변량들의 영향을 검정하기 위해서는 다항 회귀분석을 수행하며 이분형 지표의 발생에 미치는 공변량 들의 영향을 검정하기 위해서는 다항 로지스틱 회귀분석을 사용한다. 특정 이분형 변수의 시간에 따른 발생에 미치는 공변량 들의 영향을 검정하기 위해서는 생존분석을 수행한다.

특정 하위집단간의 연속형 변수의 차이는 independent t-test (혹은 정규성 검정 결과에 따라 Wilcoxon rank sum test)를 사용한다. 명목형 변수의 차이는 Chi-square test (혹은 Fishers’s Exact test)를 사용한다.

**12. 연구 대상자 보상**

○ 연구책임자는 연구대상자에게 1회 조사당 참여비(교통비)로 10만원을 제공함. 2년차에 등록된 환자의 경우 6회의 방문을 모두 완료하면 최대 10만원*6회를 지급하고, 6년차에 등록된 환자의 경우 2회의 방문을 모두 완료하면 최대 10만원*2회를 지급함. 일괄 지급이 아닌 매 방문시 지급이므로 만약 중도 탈락하는 연구대상자의 경우에도 지급에 차별이 없음

**13. 연구 대상자의 사생활 보호, 개인정보 보호 및 처리**

(1) 임상연구계획서의 숙지

○ 본 연구는 Helsinki 선언에 입각하여 연구대상자의 권리와 복지를 염두에 두고 준비된 것으로서, 연구책임자 및 담당자들이 연구계획을 정확히 분석 및 숙지하고, 임상연구 대상 연구대상자의 건강과 인권을 우선시하고, 임상연구 중 발생할 수 있는 문제점을 해결하는데 적극적으로 임한다.

(2) 임상연구계획서 준수 및 임상연구계획서 변경

○ 본 임상연구는 승인된 임상연구계획서에 따라 실시한다. 임상연구계획서의 모든 변경은 의뢰자와 논의되어야 하며, 변경계획서는 의뢰자가 작성한다. 연구자는 연구대상자에게 위해가 발생되는 것을 즉각적으로 막기 위한 경우를 제외하고는, 이러한 연구계획서 변경에 대하여 기관생명윤리위원회로부터 검토 승인을 얻기 전에는 변경된 내용을 적용해서는 안 된다. 중대한 연구계획서 위반에 대해서는 증례기록지에 기록해야 한다.

○ 만일 즉각적으로 연구대상자에게 위해가 발생하지 못하게 하기 위하여 기관생명윤리위원회의 승인을 얻기 전에 이러한 연구계획서의 변형이나 변경을 적용하게 되는 경우, 가능한 한 빨리 이러한 변형이나 변경에 대하여 기관생명윤리위원회(추후 검토 승인을 위하여), 의뢰자, 관련 부처(관련 규정에서 요구하는 경우)에 제출하여야 한다. 만일 연구계획서 변경내용이 사소한 것이면 연구자는 기관생명윤리위원회에 통보하는 것으로 충분하다. 그러나 연구디자인을 본질적으로 변경하거나 연구대상자에게 위험 가능성이 증가되는 경우라면,

1) 연구대상자 동의서를 수정하여 기관생명윤리위원회에 제출하여 검토 승인을 받아야 하고,

2) 이러한 변경으로 연구대상자들에게 영향을 주게 된다면 이미 연구에 모집된 연구대상자들로부터 새로 변경된 동의서에 다시 동의를 얻어야 하며,

3) 새로 모집하는 연구대상자들로부터는 새로운 동의서를 사용하여 동의를 얻어야 한다.

(3) 연구대상자 동의

○ 임상연구에 들어가기 전에 연구대상자에게 임상연구 내용 및 안전성에 대한 모든 사항을 설명한 후 연구대상자 자신이 자발적으로 본 연구에 참여하겠다는 서면동의서를 받고 연구에 들어간다. 연구대상자동의는 Helsinki 선언에 근거하는 윤리적 원칙에 따라야 한다.

○ 연구자는 연구참여 동의서 1부, 전문가용어가 아닌 쉽게 이해할 수 있는 용어로 임상연구에 대하여 설명해 놓은 서면 설명문 1부를 연구대상자나 법정대리인에게 제공해야 한다. 연구자는 연구대상자나 연구대상자의 대리인에게 임상연구 세부적인 사항에 대하여 질문할 수 있는 시간을 충분히 준 이후에 연구대상자나 연구대상자의 법정대리인이 동의서에 서명하고 본인이 직접 날짜를 기입하도록 해야 하고, 필요하다면 연구대상자 동의를 받을 때 함께 논의했던 자 또한 서명한다. 연구대상자나 법정대리인은 연구대상자가 연구에 참여하기 전에 서명된 동의서 1부와 서면 설명문 1부를 받아야 한다.

○ 연구대상자나 법정대리인이 문맹인 경우, 동의를 받는 전 과정동안 공정한 입회자가 입회해야 한다. 연구대상자나 법정대리인이 구두로 동의하고 서명한 후 할 수 있다면 공정한 입회자가 서면동의서에 정보가 정확했고 연구대상자나 법정대리인이 이해했으며 자유의사에 의해 동의했다고 증명하는 서명을 하고 직접 날짜를 기입해야 한다.

○ 동의서와 연구대상자나 대리인에게 제공된 기타 어떠한 정보는 연구대상자의 동의와 관련되는 중요한 정보가 새로 나오게 될 때마다 바꾸어 제공해야 하며 사용하기 전에 기관생명윤리위원회의 의견을 들어야 한다. 연구자는 연구대상자나 연구대상자의 대리인에게, 임상연구의 모든 타당한 측면과 연구대상자가 임상연구참여를 기록하기로 결정하는 의지와 관련된 새로운 정보를 완전하게 설명해 주어야 한다. 이러한 상호 의견교류에 대하여 기록해 두어야 한다.

○ 연구기관, 연구비지원기관 및 보건복지부 지정 공용기관생명윤리위원회가 실태 조사를 하는 경우에는 비밀로 유지되는 나의 개인 신상 정보를 직접적으로 열람하는 것에 동의합니다

○ 또한 본 연구는 한의약진흥원의 정부 지원 과제 특성상 추후 익명화를 통해 건강보험 청구자료 등의 2차 자료원과의 연계가 필요하여 연구정보 제 3자 제공 및 2차 연구 이용에 대한 동의도 별도로 취득한다. 2차연구 활용을 위해 제공된 연구정보는 일차 연구가 종료되는 시점부터 정보 폐기 요청 시 또는 최대 10년간 보관/관리/제공될 예정이며, 보관기간이 지난 자료 중 개인정보에 관한 사항은 「개인정보보호법」 시행령 제16조에 따라 파기한다. 수집되는 개인정보와 민감정보는 다음과 같다.

**(개인정보) 성명, 생년월일, 성별, 주소, 휴대전화 번호**

**(민감정보) 건강에 관한 정보(질병코드(KCD), 신장, 체중, 흡연, 음주, 교육, 직업, 과거력, 약물복용력, 수술력, 가족력, 병력, 활력징후, 변증진단, 검사 및 각종 설문지 결과)**

따라서, 연구자는 개인정보보호법에 의거하여 연구대상자나 법정대리인에게 연구대상자의 개인 정보에 대한 권리에 대해 숙지하도록 충분히 설명해야 한다. 연구대상자나 법정대리인은 본인의 자율적인 의지에 따라 연구대상자의 개인정보 중 본 연구에서 요구하는 문서 또는 자료를 연구의뢰자 및 관련 기관의 제 3자에게 공개하는 것에 동의해야 한다. 연구 의뢰자는 개인정보보호법에 의거하여 작성된 개인정보수집·이용·제공동의서를 각 연구기관에 연구담당자에게 제공하고 해당 연구담당자는 연구대상자나 법정 대리인에게 배부 후 동의를 득 해야 한다. 따라서, 연구정보 제 3자 제공 및 2차 연구 이용에 대한 안내문 및 동의서 1부 (이하 2차연구 이용 동의서)를 별도로 제공한다.

(4) 정확한 연구대상자의 선정

○ 본 임상연구에 앞서 충분한 조사를 통하여 연구대상자 적합여부에 대하여 철저히 평가한다.

(5) 임상연구의 진행점검

○ 임상연구책임자는 주기적으로 임상연구진행, 상황, 결과 등에 대하여 확인하며, 기관생명윤리위원회는 필요시 임상연구 진행상황에 대하여 점검을 실시한다.

(6) 임상연구 실시기관의 모니터링

○ 연구대상자의 권리와 복지보호, 보고된 임상연구 관련 자료가 근거문서와 대조하여 정확하고, 완전하며, 검증이 가능한지 여부 확인, 임상연구가 승인된 계획서, 임상연구관리기준 및 시행규칙 규정에 따라 수행되는지 확인을 위해 모니터링을 실시한다. 임상연구에 대한 모니터링은 지정한 별도의 요원이 정기적인 임상연구기관 방문 혹은 전화를 통해 실시할 수 있다. 방문 시 모니터는 기본적으로 연구대상자 기록원본, 시술관리기록, 자료보관 등을 확인한다. 또한 모니터는 임상연구 진행과정을 잘 살피고, 문제가 있을 경우 연구자와 상의한다. 본 연구의 모니터링은 주관연구기관인 원광대학교 한방병원의 연구자 및 지정하는 모니터링 담당자가 시행한다.

(7) 연구대상자의 비밀유지

○ 연구대상자의 신원을 파악할 수 있는 기록은 비밀로 보장될 것이며, 임상연구의 결과가 출판될 경우에도 연구대상자의 신원을 비밀상태로 유지한다. 구체적인 내용은 다음과 같다. 본 연구에 관련된 의뢰자, 모니터 및 점검자는 본 연구의 모니터링과 점검 및 진행사항 관리를 위한 목적으로 연구대상자의 기록을 열람할 수 있다. 연구자는 본 임상연구의 계약이 체결됨으로서 임상연구 의뢰자 또는 임상연구수탁기관의 모니터 및 점검자가 연구대상자의 차트와 증례기록서 기록을 검증하기 위하여 해당 문서를 검토하거나 복사할 수도 있음을 숙지하여야 한다. 증례기록서 등 임상연구에 관련된 모든 서류에는 연구대상자 이름이 아닌 연구대상자식별코드(일반적으로 연구대상자에 부여된 순번, 이니셜)로 기록하고 구분한다.

(8) 자료 결과 및 보고

○ 연구자는 본 연구에 참여한 개개 연구대상자별로 모든 관찰 결과 등 모든 필요한 자료를 기록할 수 있도록 디자인된 증례기록서에 적절하고 정확하게 증례내역을 작성하고 유지해야 한다.

○ 근거문서에서 유래된 것으로 증례기록서에 기재된 자료는 근거문서와 일치해야 하며, 일치하지 않는 내용에 대해서는 설명이 있어야 한다.

○ 증례기록서는 잘 알아볼 수 있도록 펜으로 작성되어야 한다. 연구대상자는 영문성명이니셜, 생년월일 및 연구대상자 식별번호로 확인하도록 한다. 요구되는 모든 정보를 증례기록서의 해당란에 기재해야 한다. 해당 정보가 없거나 적용되지 않는 항목인 경우는 그렇다고 기재하고 절대 공란으로 남겨두지 않도록 한다. 연구대상자 확인이 가능한 기록의 기밀을 유지하여, 관련 규정에 따라 연구대상자의 사생활과 비밀이 보장되어야 한다.

○ 자료 수정 시에는 잘못 기입된 부분에 한 줄을 긋고 그 옆에 다시 정확한 자료를 적은 다음, 정정한 사람의 이름 이니셜과 날짜를 기록하고, 필요한 경우 왜 수정했는지 이유를 적는다.

○ 작성 완료된 증례기록서는 연구자나 연구 참여기관에서 연구자로서의 자격이 있는 의사가 신속히 검토한 후 서명하고 날짜를 기록한다. 연구자는 변경 및 수정을 포함한 증례기록서의 사본 1부를 보관해야 한다.

(9) 기관생명윤리위원회

○ 연구를 시작하기 전 연구자는 연구계획서, 연구대상자동의서, 연구대상자모집과 관련된 자료 및 절차, 연구대상자에게 제공될 서면 설명문 등에 대하여 기관생명윤리위원회로부터 서면 승인을 받아야 한다. 연구자는 관련 규정이나 병원내 절차에 따라 임상연구 결과보고서, 최신정보, 그리고 기타정보(ex. safety updates, 변경서, 통보서)를 기관생명윤리위원회에 제공해야 한다.

**14. 연구 대상자에게 기대되는 위험과 이익**

○ 연구대상자는 본 연구 추적과정에서 설문조사를 시행하게 되며 설문조사로 인해 시간적 소요(120분 가량)가 발생할 수 있음.

○ 본 연구는 레지스트리 연구로 예상되는 위험 가능성은 매우 낮으나, 채혈 과정에서 통증, 불편감, 멍, 피로 등의 통상적 반응이 있을 수 있으며 드물게 저림, 혈종, 감염, 신경 손상 등의 이상반응이 있을 수 있음.

○ 연구대상자가 본 연구에 참여함으로서 직접적인 금전적 이익이 발생하지는 않으며, 본 연구를 통해서 해당 질환에 대한 한의 치료 레지스트리가 구축됨으로서 향후 대상 질환의 양질의 의료서비스 제공받을 수 있는 간접적인 이익이 있음.

○ 연구대상자는 프로그램 내 다양한 신체 검진을 받을 수 있음.

**15. 연구 대상자의 안전보호대책과 보상방법 등**

○ 예상치 못한 방문이 있을 경우에 방문 사유, 병력, 약물 변화, 활력징후를 증례기록지에 기록함.

○ 강제 또는 부당한 영향의 가능성을 최소화시킬 방법 : 강압의 가능성이나 부당한 영향을 최소화하기 위하여 연구대상자의 연구 참여 여부 결정은 자발적인 것이며, 연구 참여를 거부하거나 연구 기간 중 언제라도 자유롭게 참여를 중단할 수 있으며 이로 인해 다음 방문에 어떠한 불이익도 전혀 받지 않을 것이며, 언제라도 연구참여 동의를 철회할 수 있음을 알림.

○ 임상연구 배상책임 보험에 가입함.

○ 취약한 연구대상자 보호에 관한 사항

- 취약한 연구대상자가 연구에 포함되어야 하는 이유: 본 연구는 인지장애(경도인지장애, 알츠하이머병, 혈관성치매) 환자를 대상으로 환자의 진단부터 치료 및 예후까지 관리를 하는 환자레지스트리(등록)연구 이므로, 일부 제한된 동의 능력을 가진 성인이 포함된다.

- 연구대상자에게 대한 위험을 최소화하기 위한 절차: 연구 참여에 대한 동의 능력을 반복적으로 평가하고, 언제든지 연구의 지속과 중단을 결정할 수 있다는 사항, 연구 참여 중단으로 인한 불이익이 없다는 사항을 반복적으로 알린다.

- 연구대상자의 동의 능력 평가에 대한 계획: 1) 연구대상자가 연구와 관련된 정보를 이해하는지 여부, 2) 연구에 대한 정보를 논리적으로 다룰 수 있는지 여부, 3) 연구에 참여하고 싶어하는지 아닌지에 관한 자신의 선택을 명확하게 의사표현하는 능력이 있는지 여부 등을 종합적으로 판단한다.

- 대리인의 동의를 포함할 계획: 위 평가에 근거하여 연구참여에 대한 동의가 어렵다고 판단되면, 연구 대상자 본인의 서면 승낙 및 법정대리인의 동의를 받는다.

- 적절한 경우에 연구대상자의 동의를 위한 계획 : 위 동의 능력 평가에 근거하여 충분한 동의 능력이 있다고 판단되는 경우 연구대상자 본인의 동의를 받는다.

**16. 자료의 보관**

○ 본 연구 중 수집·취득되는 모든 자료는 연구 및 결과보고서 작성 이외의 다른 목적으로는 사용하지 않음. 연구대상자의 자료는 분실 및 도난을 방지하기 위해 자물쇠를 사용하여 보관하고, 보관책임자는 임정태 교수로 하며, 연구 종료 후에는 원광대학교 한방병원 임상연구센터의 연구실 문서보관함에 분실 및 도난을 방지하기 위해 자물쇠를 사용하여 3년간 보관 후 파기함. 다만, 의뢰자가 필요성이 있다고 판단한 경우에는 보존기간을 연장할 수 있음.

○ 본 연구 중 수집·취득되는 자료 및 기록의 폐기 절차는 전자적 파일 형태로 기록·저장되는 정보는 기록을 재생할 수 없도록 파기하며, 종이 문서에 기록·저장되는 정보는 분쇄한 후 용해하여 파기함.

**17. 참고문헌**
